# Global prevalence of impostor phenomenon in medical students: A systematic review and meta-analysis

**DOI:** 10.1371/journal.pone.0351713

**Published:** 2026-08-26

**Authors:** Víctor Juan Vera-Ponce, Christian Humberto Huaman-Vega, Fiorella E. Zuzunaga-Montoya, César Iván Guerrero Uceda, Darwin A. León-Figueroa, Oriana Rivera-Lozada, Edwin Aguirre-Milachay, Carmen Inés Gutierrez De Carrillo, Mario J. Valladares-Garrido

**Affiliations:** 1 Instituto de Investigación de enfermedades Tropicales, Universidad Nacional Toribio Rodríguez de Mendoza de Amazonas (UNTRM), Amazonas, Perú; 2 Facultad de Medicina (FAMED), Universidad Nacional Toribio Rodríguez de Mendoza de Amazonas (UNTRM), Amazonas, Perú; 3 Instituto de Investigación de Salud Integral Intercultural, Universidad Nacional Toribio Rodríguez de Mendoza de Amazonas (UNTRM), Amazonas, Perú; 4 Universidad Continental, Lima, Perú; 5 Escuela de Medicina Humana, Universidad Señor de Sipán, Chiclayo, Peru; 6 Unidad de Posgrado y Especialización, Tecnologías Avanzadas para la Salud con enfoque en Inteligencia Artificial, Bioinformática y TIC, Universidad Peruana Cayetano Heredia, Lima, Peru; 7 EpiHealth Research Center for Epidemiology and Public Health, Lima, Peru; 8 Hospital Nacional Almanzor Aguinaga Asenjo, EsSalud, Chiclayo, Peru; 9 Oficina de Inteligencia Sanitaria, Red Prestacional EsSalud Lambayeque, Chiclayo, Peru; Fundación Universitaria del Área Andina, COLOMBIA

## Abstract

**Introduction:**

Impostor phenomenon (IP) is a psychological phenomenon in which successful individuals fail to internalize their achievements and fear being exposed as “incompetent.” Medical students may be particularly vulnerable to IP because medical training combines repeated high-stakes assessment, competitive peer comparison, hierarchical clinical supervision, and rapid professional identity transition, potentially affecting psychological well-being and academic performance.

**Objective:**

To estimate the global prevalence of IP in pre-licensure medical students and analyze differences by sex, academic stage, and geographic region.

**Methods:**

A systematic search was conducted in MEDLINE/PubMed, Scopus, Web of Science, and EMBASE, including studies published between January 1, 2000, and June 30, 2025. Observational studies reporting IP prevalence in pre-licensure medical students using validated instruments were selected. Pooled prevalences were calculated using random-effects meta-analysis, with subgroup analyses and meta-regression by publication year.

**Results:**

Thirty-five studies were included for qualitative synthesis, with 26 studies (n = 9,110 students) employing the Clance Impostor Phenomenon Scale (CIPS) included in the primary meta-analysis. The global CIPS-based prevalence was 54.2% (95% CI: 47.0%−61.3%) with high heterogeneity (I² = 98%). Studies using probabilistic sampling (n = 4) reported 36.5% prevalence, while non-probabilistic sampling (n = 22) yielded 57.5%. Prevalence was similar between women (50.5%) and men (47.1%), and between preclinical (48.9%) and clinical (46.8%) students. The highest rates were observed in the United Arab Emirates (89.5%), Canada (75.8%), and the United Kingdom (66.0%), while Peru presented the lowest estimate among CIPS studies (30.6%). Meta-regression showed no significant temporal trend from 2018–2024 (β = 0.0221, p = 0.1747).

**Conclusions:**

IP was frequent among pre-licensure medical students, although pooled estimates should be interpreted cautiously because heterogeneity was substantial and prevalence varied by sampling method and geographic context. These findings support the need for standardized measurement, probabilistic sampling when feasible, longitudinal research, and rigorously evaluated interventions addressing both individual coping and institutional educational practices.

## Introduction

Impostor phenomenon (IP) is a psychological phenomenon wherein successful individuals fail to internalize their achievements and fear being exposed as “incompetent” [1]. We use the term “impostor phenomenon” rather than “impostor syndrome” because IP is not a formal psychiatric diagnosis and the term “syndrome” may pathologize a context-sensitive experience; the term “impostor syndrome” is retained only when referring to terminology used in original studies, search strategies, or specific instruments [1,2]. Conceptually, IP can be understood as a multidimensional self-evaluative pattern that may show trait-like persistence in some individuals but may also be activated or intensified by specific contexts, particularly high-stakes evaluative environments, transitions in professional identity, and repeated comparison with high-performing peers. This phenomenon presents particularly high prevalence rates in university and health-professional populations, ranging from 30% to 70% [3,4]. The consequences described in student and health-professional populations include increased risk of anxiety, depression, and burnout, contributing to deterioration of psychological well-being, professional exhaustion, and suicidal ideation [4,5]. Affected students typically report low self-esteem, decreased academic performance, and recurrent thoughts about dropping out, compromising their long-term professional development [5].

Medical students represent a particularly vulnerable population to this phenomenon, with reported prevalence rates between 22% and 89%, reaching over 75% in competitive, high-stakes, and clinically supervised educational contexts [6]. Medical training exposes students to recurrent summative examinations, competitive peer comparison, hierarchical feedback, early patient-care responsibility, and rapid professional identity formation. These features may make medical training a setting in which self-doubt is not merely an individual vulnerability but also a response to repeated evaluation, uncertainty, and comparison during professional socialization. Although prior reviews have examined physicians and physicians in training [6–8], no dedicated global meta-analysis had synthesized IP prevalence specifically among pre-licensure medical students across instruments, sampling strategies, academic stages, and regions. This gap justifies a dedicated review that estimates the global scope of IP in this population while accounting for methodological and contextual sources of heterogeneity.

Therefore, this systematic review and meta-analysis aims to estimate the global prevalence of IP in pre-licensure medical students, providing a consolidated rate that reflects its scope across different medical educational contexts. Additionally, differences by sex, academic stage (preclinical versus clinical), and geographic region will be explored, allowing for the identification of differentiated patterns specific to medical education and a better understanding of influential contextual factors. By clarifying the magnitude of the problem in this group, the findings can guide interventions adapted to the medical educational setting, from coping workshops to institutional student wellness programs. The results can inform the development of systemic interventions in medical schools, contributing to improved mental health and academic performance of future physicians.

## Methodology

### Study design

A systematic review and meta-analysis was conducted to estimate the global prevalence of IP in pre-licensure medical students. This review followed the PRISMA (Preferred Reporting Items for Systematic Reviews and Meta-Analyses) guidelines [9] (S1 Table), adapted for prevalence studies according to current methodological recommendations [10,11], to ensure transparency, reproducibility, and quality of findings. The review protocol was not prospectively registered in PROSPERO, and no registration number is available.

### Search strategy

A comprehensive search was performed in the electronic databases MEDLINE/PubMed, Scopus, Web of Science (including the SciELO catalog), and EMBASE. These databases were selected because they provide broad biomedical, epidemiological, multidisciplinary, and medical-education coverage and are commonly used in systematic reviews [12,13]. PsycINFO was not searched because of limited institutional access. We acknowledge that this may have led to missed psychology-focused records. To mitigate this limitation, we searched MEDLINE/PubMed, EMBASE, Scopus, and Web of Science, complemented by manual reference checking. Previous methodological work supports the high retrieval value of combining biomedical databases with multidisciplinary citation databases, although omission of a discipline-specific psychology database remains a limitation [13]. Specific search strategies were used for each database, including MeSH terms and keywords combined using Boolean operators (“AND”, “OR”). Terms employed included: “impostor syndrome”, “impostor phenomenon”, “medical students”, “Clance Impostor Phenomenon Scale”, and “prevalence”.

The search covered the period from January 1, 2000, to June 30, 2025, without language restrictions. The year 2000 was selected to focus on contemporary medical education contexts and studies using modern validated psychometric instruments for IP assessment. The complete and detailed search strategy for each database is available in S2 Table. A manual search of references from included studies was conducted to identify potentially eligible articles not retrieved in the primary search.

### Selection criteria

Observational studies reporting IP prevalence in pre-licensure medical students were included, regardless of whether the medical program was entered directly after secondary education or after a prior undergraduate degree, and without restrictions by sex, geographic location, or language. Cross-sectional designs, cohort studies, and secondary analyses presenting prevalence estimates using validated psychometric instruments, such as the Clance Impostor Phenomenon Scale (CIPS) [1,2], Young Impostor Scale (YIS), Leary Impostor Scale, or other internationally recognized scales, were accepted. Studies were required to explicitly report the number of cases and total sample size, or sufficient information to calculate them. Studies including mixed populations were eligible for qualitative synthesis when medical students were part of the sample and IP assessment was clearly reported; they were included in quantitative synthesis only when the numerator and denominator for medical students could be extracted separately. No minimum proportion of medical students was required when medical-student-specific prevalence data were available.

Letters to the editor, commentaries, case reports, qualitative studies, previous bibliographic or systematic reviews, bibliometric studies, and those evaluating IP through subjective self-report without validated instruments were excluded. Studies conducted exclusively in non-medical populations, medical residents, licensed healthcare professionals, or those considering the phenomenon exclusively in workplace contexts were also excluded.

### Study selection process

After applying the search strategy, all retrieved records were imported into Rayyan QCRI software for automated duplicate removal. Subsequently, two independent reviewers (VJV-P and FEZ-M) blindly and in parallel evaluated titles and abstracts according to previously defined inclusion criteria. Potentially eligible articles underwent full-text review to confirm final eligibility. Discrepancies included disagreements about population eligibility, instrument validity, extractability of medical-student-specific prevalence, and compatibility of IP thresholds for quantitative synthesis.

Discrepancies between reviewers were resolved through discussion and, if disagreements persisted, a third reviewer intervened (MJV-G). This process ensured consistent and rigorous selection criteria across all screening phases. Formal inter-rater reliability statistics were not calculated because individual pre-consensus screening decisions were not retained in a format suitable for reliable estimation. The complete selection process was documented using a PRISMA flow diagram.

### Data extraction

Two reviewers (CHH-V and FEZ-M) performed data extraction independently using a standardized template in Microsoft Excel 2023, previously piloted. From each study, the following data were collected: first author’s name, publication year, country or region of study, study design, collection period, sample size, population characteristics (sex as reported by the original studies, average age, and academic year or academic stage), sampling method, instrument used for IP assessment, defined cut-off point, and overall prevalence (with or without confidence interval) and by subgroups (sex, academic stage, regions). All extracted data were cross-checked between both reviewers, and, in case of differences, the original texts were jointly reviewed. Errors or inconsistencies were corrected by consensus.

### Risk of bias assessment

Methodological quality was assessed using the JBI critical appraisal checklist for studies reporting prevalence data, as described by Munn et al. [10]. This tool includes nine items evaluating sampling frame, participant recruitment, sample size, description of subjects and setting, data coverage, validity of measurement, standardization of measurement, statistical analysis, and response-rate handling. Each item was rated as “Yes” (1 point) or “No/Unclear” (0 points), with total scores ranging from 0 to 9. Studies were classified as low risk of bias (7–9), moderate risk (4–6), or high risk (0–3).

Two reviewers (VJV-P and FEZ-M) independently assessed each study, with discrepancies resolved by consensus or by a third reviewer (MJV-G). Risk-of-bias findings were considered in the qualitative interpretation of results. Sensitivity analyses based on risk-of-bias level were planned but were not performed because all included studies were classified as low risk of bias.

### Statistical analysis

A prevalence meta-analysis stratified by measurement instrument was performed, analyzing studies separately according to the scale used to preserve conceptual homogeneity in construct measurement. The CIPS-based meta-analysis was considered the primary quantitative synthesis because CIPS is the most frequently used IP measure in the included literature, uses a 20-item format, and provides conventional severity thresholds that allow more consistent prevalence synthesis. Studies using alternative instruments, such as the YIS or Leary Impostor Scale, were summarized separately to avoid combining non-equivalent measurement constructs. The YIS analysis was considered exploratory. All analyses were conducted using R software (version 4.1.0) with the “meta” and “metafor” packages, applying random-effects models specifically chosen to accommodate anticipated between-study heterogeneity. This model assumes that true prevalence varies across populations, which is consistent with proportional meta-analysis guidance and the expected variability in IP manifestation across medical education contexts [14]. Prevalence estimates and their 95% confidence intervals were calculated using the Freeman-Tukey double arcsine transformation to stabilize variance, while confidence intervals were obtained using the Wilson Score method.

Statistical heterogeneity between studies was assessed using Cochrane’s Q test and the I² statistic. While I² values above 75% conventionally indicate substantial heterogeneity, we interpreted this metric within the context of prevalence meta-analyses, where high I² values are common and should be interpreted with caution rather than as an automatic reason to avoid synthesis [14,15]. To systematically explore sources of anticipated heterogeneity, we conducted pre-specified subgroup analyses according to: (a) sampling method (probabilistic versus non-probabilistic), recognizing this as a critical methodological moderator; (b) sex; (c) academic stage; and (d) geographic region. Academic stage was operationalized according to the definitions reported by each study. When not explicitly defined, years 1–3 were classified as preclinical and year 4 onward as clinical, recognizing that this classification varies across medical education systems. Meta-regression was performed with publication year as the independent variable to evaluate temporal trends in reported prevalence and assess whether awareness of the phenomenon has influenced reporting patterns. Meta-regression by publication year was restricted to studies included in the primary CIPS-based quantitative synthesis; therefore, the temporal range for this analysis was 2018–2024. Small-study effects and reporting bias were explored using funnel plot inspection and Egger’s test when the number of studies was sufficient for such assessment.

For the graphical presentation of results, forest plots were organized both chronologically and by region to visualize patterns of heterogeneity. Bubble plots illustrated the relationship between publication year, sample size, and estimated prevalence, allowing visual assessment of potential small-study effects and temporal trends. These visualizations serve to transparently communicate both the central estimates and the substantial variability inherent in this global synthesis.

## Results

### Study selection

**Fig 1:**
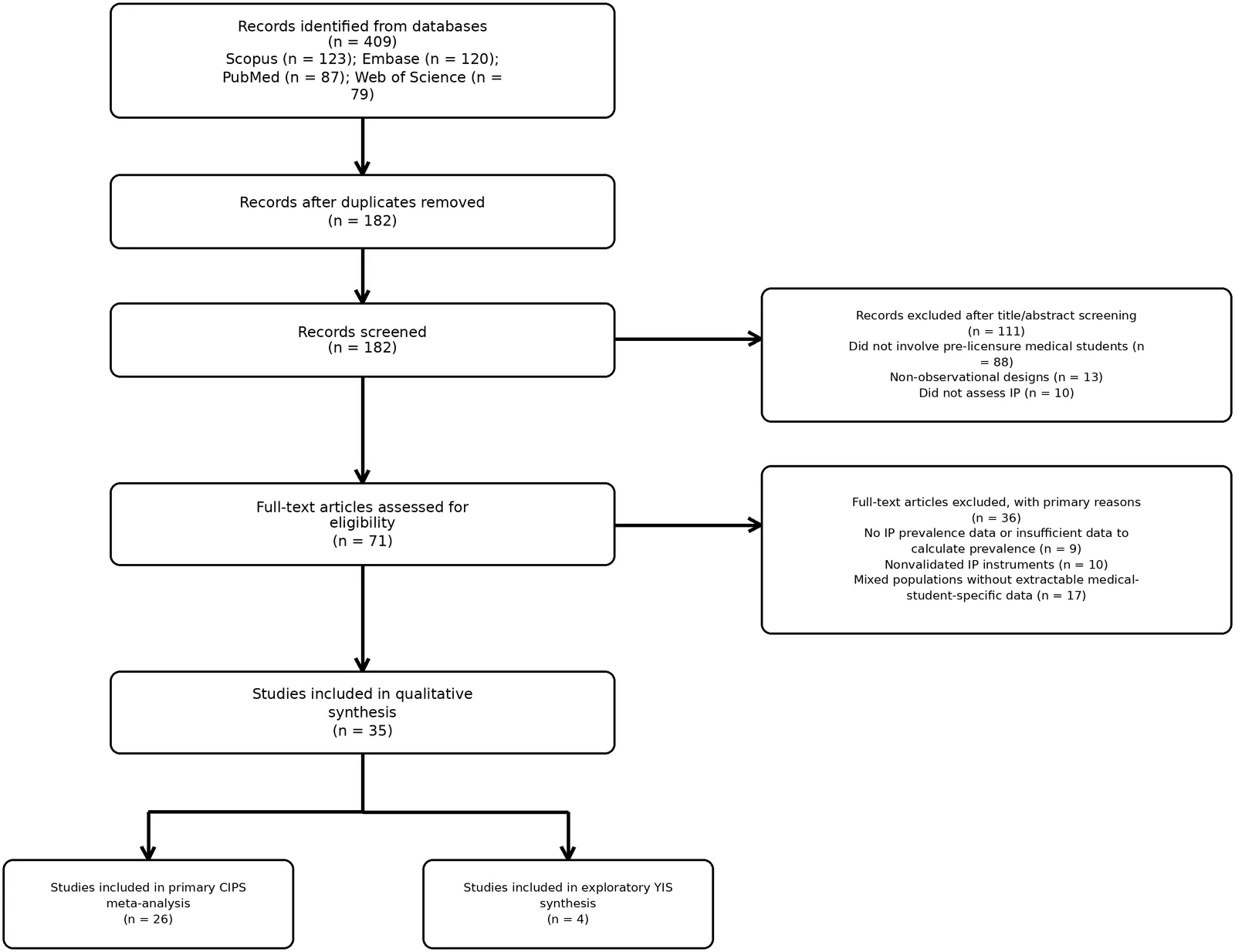
Flowchart of study selection.

The study selection process is illustrated in Fig 1. The initial search in electronic databases identified 409 records: Scopus (n = 123), Embase (n = 120), PubMed (n = 87), and Web of Science (n = 79). After duplicate removal, 182 unique records were screened. Of these, 111 records were excluded during title and abstract screening because they did not involve pre-licensure medical students (n = 88), had non-observational designs (n = 13), or did not assess IP (n = 10). Seventy-one full-text articles were assessed for eligibility. Of these, 36 were excluded according to the primary reason for exclusion: failure to report IP prevalence data or sufficient data to calculate prevalence (n = 9), use of nonvalidated instruments for IP assessment (n = 10), and mixed populations without extractable medical-student-specific data (n = 17). Finally, 35 studies met all eligibility criteria and were included in the qualitative synthesis [16–50]. Of these, 26 studies using CIPS with comparable thresholds were included in the primary quantitative meta-analysis. The remaining nine were not included in the primary CIPS meta-analysis because five used YIS, one used the Leary Impostor Scale, and three CIPS reports did not provide data in a format suitable for primary pooling. Four YIS studies with comparable thresholds were analyzed in an exploratory synthesis; the remaining YIS study was summarized qualitatively because its outcome definition was not directly comparable.

### Qualitative synthesis: Characteristics of included studies

A total of 35 studies published between 2016 and 2024 were included [16–50], with a notable increase in scientific production from 2020 onwards, which accounted for 83% of the studies (n = 29). Most articles were published in English (97%), while only one was identified in Portuguese (Brazil). This coverage allowed integration of a multi-regional perspective of the phenomenon across diverse academic and cultural realities.

Cross-sectional designs predominated (97%, n = 34), with only one study implementing a longitudinal design (Rosenthal, 2021) [19]. Most studies used non-probabilistic sampling (85.7%, n = 30), mainly through convenience or voluntary self-selection. Five studies implemented probabilistic sampling, including simple or stratified random sampling (Egwurugwu, 2018; Alsaleem, 2021; Elnaggar, 2023; Al Lawati, 2023; and Vilchez-Cornejo, 2023) [18,25,26,28,39]. Among the 26 CIPS studies included in the primary meta-analysis, four used probabilistic sampling and 22 used non-probabilistic sampling. Sample sizes showed high variability, ranging from 49 to 2,231 students, with a median of 256 participants and a mean of approximately 360 students per study (Table 1).

**Table 1:** Main characteristics of the selected studies.

First author (Year)	Year	Country	Sampling type	Sample size	Selection criteria	Age	% Women	Instrument used	Instrument validation	Risk of bias analysis
**Villwock (2016)**	2016	USA	Non-probabilistic	138	Medical students in training included	25–30	56.20%	YIS – 8 items	Yes. Validated scale with references to previous studies. Alpha was not reported in this study.	7 points (low risk)
**Qureshi (2017)**	2017	Pakistan	Non-probabilistic	143 (response rate 95.33%)	Final year MBBS students, private medical college Lahore	24.08 ± 1.051 years	58.70%	YIS – 8 items	Yes. Validated scale with references to previous studies. Alpha was not reported in this study.	7 points (low risk)
**Ikbaal (2018)**	2018	Malaysia	Non-probabilistic	256	Medical students at Melaka-Manipal Medical College	Not specified	60.90%	CIPS – 20 items	Yes. Validated scale with references to previous studies. Alpha was not reported in this study.	7 points (low risk)
**Egwurugwu (2018)**	2018	Nigeria	Probabilistic	200	Pre-licensure medical students, Imo State University	21.35 ± 3.01 years (range 17–36)	48%	CIPS – 20 items	α=0.83 for internal consistency	8 points (low risk)
**Maqsood (2018)**	2018	Pakistan	Non-probabilistic	189 (response rate 94.5%)	Medical students all years, Nishtar Medical College Multan	Not specified	35.97%	CIPS – 20 items	α=0.90 reported	7 points (low risk)
**Holliday (2019)**	2019	USA	Non-probabilistic	485	Harvard Medical School and Harvard School of Dental Medicine students (all years)	25 years (range 21–34)	52%	CIPS – 20 items	Validated scale (Clance 1985); reliability not reported in this study	7 points (low risk)
**Mascarenhas (2019)**	2019	India	Non-probabilistic	150	Medical interns at Goa Medical College	Mean: 22.72 ± 0.72 years	62%	CIPS – 20 items	Yes. Validated scale with references to previous studies. Alpha was not reported in this study.	7 points (low risk)
**Levant (2020)**	2020	USA	Non-probabilistic	112	Students transitioning from the preclinical to the clinical phase included	25.8	59%	CIPS – 20 items	Yes. Cronbach’s alpha = 0.92	7 points (low risk)
**Shreffler (2020)**	2020	USA	Non-probabilistic	233	Medical students (M2-M4) at University of Louisville who had completed Step 1	Not reported	45.60%	CIPS – 20 items	Previously validated; Cronbach’s α = 0.938	7 points (low risk)
**Levant (2020)**	2020	USA	Non-probabilistic	127	Third-year medical students at University of Kansas School of Medicine	Not reported	53.10%	CIPS – 20 items	Cronbach’s α = 0.938	7 points (low risk)
**Lee (2020)**	2020	USA	Non-probabilistic	959	Graduate students and professionals in STEM and medicine (48 US states)	Not reported (range 20–61)	79.80%	CIPS – 20 items	Cronbach’s α = 0.91	7 points (low risk)
**Alsaleem (2021)**	2021	Saudi Arabia	Probabilistic	573	Medical students included, both sexes	21	46.80%	YIS – 8 items	NR	9 points (low risk)
**Rosenthal (2021)**	2021	USA	Non-probabilistic	257	Medical students included (pre- and post-first year)	22	50%	CIPS – 20 items	Yes. Cronbach’s alpha = 0.84	7 points (low risk)
**Brennan-Wydra (2021)**	2021	USA	Non-probabilistic	226	Medical students at Yale School of Medicine (all years)	Not reported	53.10%	Leary Impostor Scale – 7 items	Cronbach’s α = 0.93	7 points (low risk)
**Camara (2022)**	2022	Brazil	Non-probabilistic	425	Students from a private university in Brazil included, from the first to the eighth semester	23	36.20%	CIPS – 20 items	Yes. Validated scale with references to previous studies. Alpha was not reported in this study.	7 points (low risk)
**Mashhadi (2022)**	2022	Pakistan	Non-probabilistic	399	Students without previous psychiatric disorders included	20–22	36.30%	CIPS – 20 items	Yes. Validated scale with references to previous studies. Alpha was not reported in this study.	7 points (low risk)
**Shill-Russell (2022)**	2022	USA	Non-probabilistic	600	Those who took classes from 2020 to 2023 included	NR	50.20%	YIS – 8 items	Yes. Cronbach’s alpha = 0.74	7 points (low risk)
**Naser (2022)**	2022	Bahrain	Non-probabilistic	290	Those at the international university in the Middle East included	19	58.30%	CIPS – 20 items	Yes. Cronbach’s alpha = 0.87	7 points (low risk)
**Campos (2022)**	2022	Brazil	Non-probabilistic	425	Pre-licensure medical students (pre-internship years 1–4) at Christus University Center who provided informed consent	23.03 ± 5.0 years (mean ± SD)	36.20%	CIPS – 20 items	Brazilian Portuguese adaptation by Bezerra et al. (2021); Cronbach’s α = 0.938	7 points (low risk)
**Neufeld (2022)**	2022	Canada	Non-probabilistic	277 (response rate 19.1%)	Medical students from 3 Canadian universities (Saskatchewan, Alberta, Calgary)	Not specified	64.30%	CIPS – 20 items	α=0.90	7 points (low risk)
**Shinawatra (2023)**	2023	Thailand	Non-probabilistic	228	Students in clinical rotation; no explicit exclusion criteria	21–25	59.60%	CIPS – 20 items	Yes. Thai validation (translation-back translation, pilot test). Cronbach’s alpha = 0.915. Confirmatory factor analysis performed.	7 points (low risk)
**Elnaggar (2023)**	2023	Saudi Arabia	Probabilistic	165	Students from Jouf University (stratified by year and sex)	18–24	52.10%	CIPS – 20 items	Yes. Validated scale with references to previous studies. Alpha was not reported in this study.	8 points (low risk)
**Al Lawati (2023)**	2023	Oman	Probabilistic	276	Medical students from Sultan Qaboos University	21.3	65.90%	CIPS – 20 items	Yes. Validated scale. Cronbach’s alpha is between 0.92 and 0.96.	8 points (low risk)
**Rice (2023)**	2023	USA	Non-probabilistic	278	Enrolled students: those providing incomplete data were excluded	NR	63%	CIPS – 20 items	Yes. Validated scale. Cronbach’s alpha is between 0.86 and 0.88.	7 points (low risk)
**Vilchez-Cornejo (2023)**	2023	Peru	Probabilistic	2,231	Students from first to sixth year included no psychiatric history	19–23	54.30%	CIPS – 20 items	Yes. Cronbach’s alpha = 0.91	9 points (low risk)
**Franchi (2023)**	2023	United Kingdom	Non-probabilistic	191	Medical students	21.1	71.20%	CIPS – 20 items	Yes. Cronbach’s alpha = 0.90	7 points (low risk)
**Sawant (2023)**	2023	India	Non-probabilistic	416	Those enrolled in the medical study program included	20.5	39.70%	CIPS – 20 items	Yes. Cronbach’s alpha = 0.96	7 points (low risk)
**Khalil (2023)**	2023	Saudi Arabia	Non-probabilistic	300	Those at King Saud bin Abdulaziz University for Health Sciences included both sexes	21.4	79.30%	CIPS – 20 items	Yes. Cronbach’s alpha > 0.90	7 points (low risk)
**Clark (2024)**	2024	USA	Non-probabilistic	89	All years, a single university	NR	NR	CIPS – 20 items	Yes. Validated scale with references to previous studies. Alpha was not reported in this study.	7 points (low risk)
**Priya (2024)**	2024	India	Non-probabilistic	249	Without mental disorders or medication	NR	57%	CIPS – 20 items	Yes. Validated scale with references to previous studies. Alpha was not reported in this study.	7 points (low risk)
**Diaconescu (2024)**	2024	Romania	Non-probabilistic	331	Students without current somatic or psychiatric morbidity	21.2	69.20%	CIPS – 20 items	Yes. Cronbach’s alpha between 0.85 and 0.9	7 points (low risk)
**Alzufari (2024)**	2024	United Arab Emirates	Non-probabilistic	399	Without chronic diseases or medications with psychiatric effects	2	64.70%	CIPS – 20 items	Yes. Cronbach’s alpha between 0.92 and 0.96	7 points (low risk)
**Kristoffersson (2024)**	2024	Sweden	Non-probabilistic	457	Students from Umeå University included, from clinical and preclinical semesters	NR	62.60%	CIPS – 20 items	Yes. Validated scale with references to previous studies. Alpha was not reported in this study.	7 points (low risk)
**Wrench (2024)**	2024	USA	Non-probabilistic	49	First-year students included, as volunteers	NR	52%	YIS – 8 items	Yes. Validated scale with references to previous studies. Alpha was not reported in this study.	7 points (low risk)
**Buathong (2024)**	2024	Thailand	Non-probabilistic	272	Those without a previous mental health diagnosis included	21.6	59.60%	CIPS – 20 items	Yes. Cronbach’s alpha = 0.946	7 points (low risk)

CIPS, Clance Impostor Phenomenon Scale; YIS, Young Impostor Scale; NR, not reported; IP, impostor phenomenon. Risk of bias was assessed using the JBI/Munn prevalence checklist. Scores range from 0 to 9; low risk = 7–9, moderate risk = 4–6, high risk = 0–3.

Studies primarily included pre-licensure medical students, covering from the first to the sixth year of medical school and including, in some cases, clinical cycles and specific transition phases. The average age of participants ranged from 18 to 30 years, with the most frequent participation from students between 20 and 23 years old. Most studies included participants of both sexes, with female proportions ranging from 35.97% to 88.9%, showing predominant female representation in most samples (median 58.3%). Sex distribution was not reported in Clark (2024) [32]. No study focused exclusively on rural or urban populations, and few provided data on socioeconomic status (Table 1).

IP assessment was conducted using validated psychometric instruments. The CIPS-20 was the most frequently used instrument for IP assessment. Five studies used the YIS as an alternative measure (Villwock, 2016; Qureshi, 2017; Alsaleem, 2021; Shill-Russell, 2022; Wrench, 2024) [16,18,22,37,40], and one study used the Leary Impostor Scale (Brennan-Wydra, 2021) [48]. For the primary meta-analysis, only the 26 studies using CIPS with clear prevalence data and comparable thresholds were included to ensure methodological consistency. The most common cut-off point for identifying threshold-defined IP was ≥ 62 on CIPS, although some studies employed severity classifications (moderate, frequent, intense). All instruments demonstrated acceptable to high internal consistency, with Cronbach’s alpha values ranging from 0.74 to 0.96 when reported (n = 23 studies), while 12 studies did not report alpha values in their publications. Local validations or cultural adaptations were reported for Thailand, Brazil, and several other countries (Table 1).

### Risk of bias assessment

Risk of bias assessment using the Munn et al. tool was conducted for all 35 studies included in the qualitative synthesis (S3 Table). All studies were classified as low risk of bias, with scores ranging from 7 to 9 out of 9 possible points. Across the 26 studies included in the primary CIPS-based meta-analysis, 22 used non-probabilistic sampling and four used probabilistic sampling. The main methodological limitations were non-probabilistic recruitment and response rates that were either not reported or not accompanied by an assessment of non-response bias, such as comparison between responders and non-responders or discussion of how low participation could affect prevalence estimates. All studies used validated IP instruments, including CIPS, YIS, or the Leary Impostor Scale, and provided operational definitions for IP assessment.

All included studies met criteria for using validated instruments and providing operational case definitions with established or explicitly reported thresholds. Studies consistently demonstrated adequate sample sizes, detailed participant descriptions, and appropriate statistical analyses. Risk-of-bias sensitivity analyses were not conducted because no study was classified as moderate or high risk of bias.

Reporting bias assessment through funnel plot analysis (S1 Fig) revealed an asymmetric distribution of studies around the pooled estimate. The plot shows a concentration of studies near the apex with relatively small standard errors, but notable dispersion at the base where larger standard errors are present. Several studies appear as outliers, particularly those reporting very high (>0.9 transformed proportion) or low (<0.6 transformed proportion) prevalences. Egger’s test for funnel plot asymmetry yielded a non-significant result (p = 0.3456), suggesting no strong statistical evidence of publication bias. However, the visual asymmetry and clustering of studies around higher prevalence values (0.8–0.9 on the Freeman-Tukey scale) indicate potential small-study effects or genuine heterogeneity rather than classic publication bias. The absence of studies in certain areas of the plot, particularly in the lower-left quadrant, may reflect either unpublished negative findings or true variation in prevalence across different populations and contexts.

### Quantitative synthesis: CIPS-based IP prevalence

To ensure methodological consistency and interpretability, only studies using the CIPS with a threshold of ≥62 points or equivalent categorizations for moderate-to-severe IP were included in the primary CIPS-based meta-analysis. Studies employing alternative instruments such as the YIS were analyzed separately, and CIPS studies with missing or non-pooling-compatible data were summarized qualitatively. Minor variations in cut-off points (≥61 or ≥63) were treated as comparable because they fall within the same conventional CIPS severity range and do not represent meaningfully different categories on the 20-item scale [1,2].

**Fig 2:**
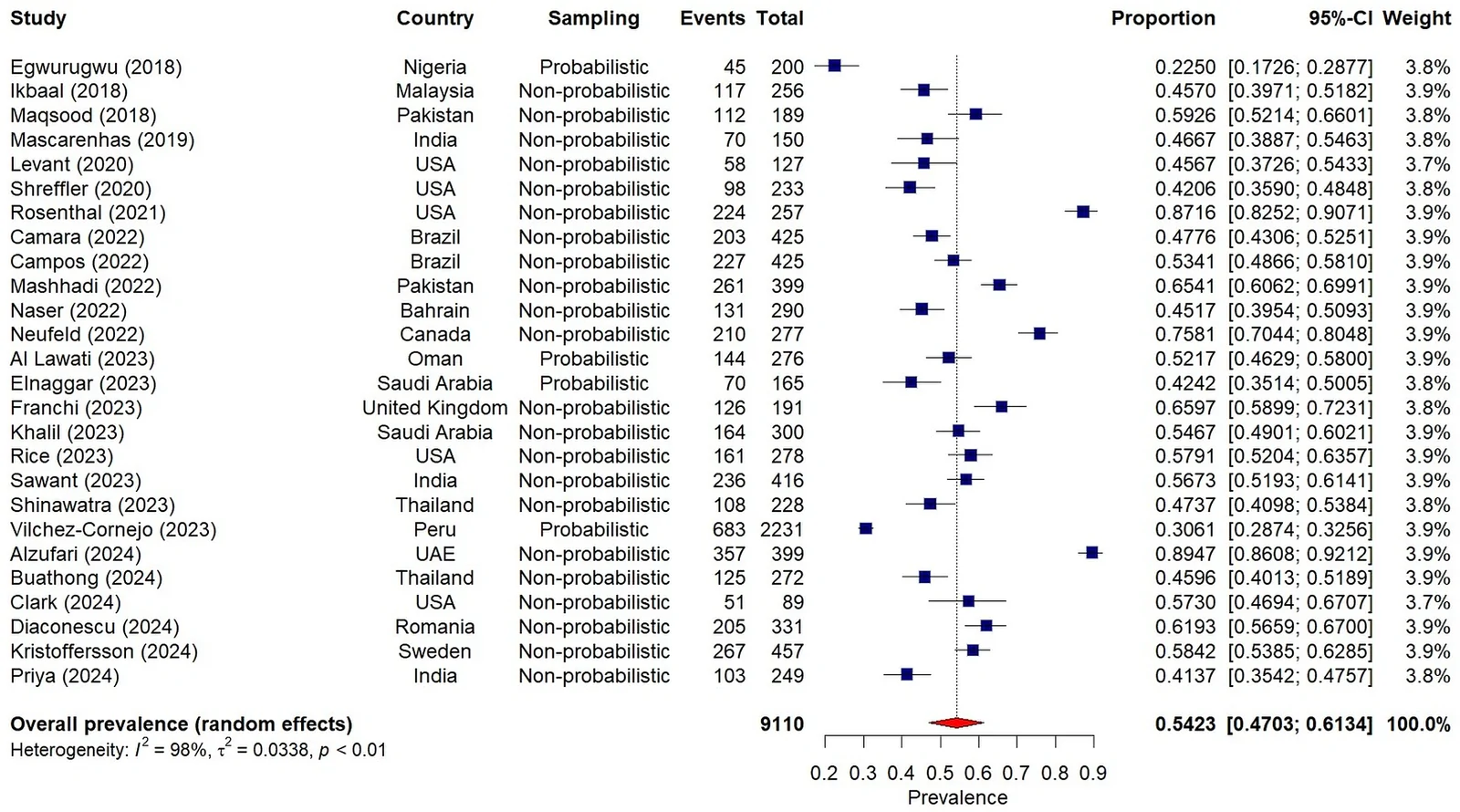
Forest plot of IP prevalence in medical students, assessed using CIPS.

A random-effects meta-analysis was performed on 26 studies comprising 9,110 medical students. The pooled global prevalence was 54.23% (95% CI: 47.03%–61.34%). Heterogeneity between studies was substantial (I² = 98%, τ² = 0.0338, p < 0.01), justifying the use of the random-effects model and caution in interpreting the pooled estimate as a single universal rate (Fig 2).

The highest prevalence was observed in Alzufari et al. (2024) [35], conducted in the United Arab Emirates, with 89.5% (95% CI: 86.1%–92.1%), while the lowest was reported by Vilchez-Cornejo et al. (2023) in Peru [28], with 30.6% (95% CI: 28.7%–32.6%). Eighteen of the 26 studies (69.2%) reported prevalences exceeding 50%, including studies from the USA, Pakistan, India, Brazil, Saudi Arabia, Thailand, and Romania. The remaining eight studies reported prevalences ranging from 30.6% to 47.8% (Fig 2).

### Exploratory synthesis: YIS-based IP prevalence

**Fig 3:**
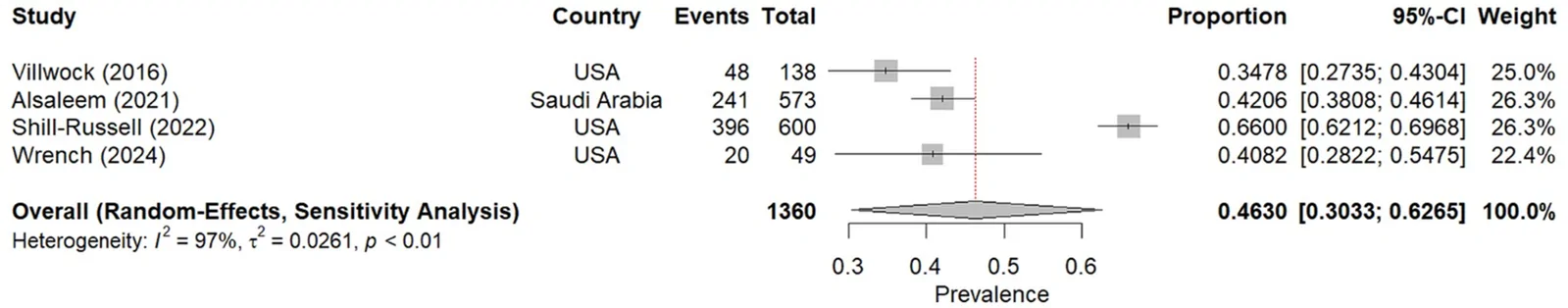
Forest plot of IP prevalence in medical students, assessed using YIS.

For this exploratory synthesis, only studies that evaluated IP using the YIS and reported comparable dichotomous thresholds were considered. Four studies were included, all with uniform cut-off points to define threshold-defined impostor symptoms (≥5 points on YIS) [16,18,22,37]. Qureshi et al. [40] was summarized qualitatively rather than pooled because its outcome definition was not directly comparable with the dichotomous YIS threshold used in the exploratory synthesis. These studies, conducted in the USA and Saudi Arabia between 2016 and 2024, comprised 1,360 medical students (Fig 3).

According to the random-effects model, the combined prevalence of IP assessed by YIS was 46.3% (95% CI: 30.3%–62.7%). Heterogeneity between studies was high (I² = 97%, p < 0.01). Individual estimates varied between 34.8% in the survey by Villwock (2016) [16] and 66.0% in the study by Shill-Russell (2022) [22], conducted in a large sample of medical students in the USA. The statistical weight of each study ranged from 22.4% to 26.3%. These results provide an exploratory prevalence estimate based on an alternative instrument to CIPS and should be interpreted separately from the primary CIPS-based synthesis (Fig 3).

### Subgroup and sensitivity analyses of CIPS-based IP prevalence

Sensitivity analyses were conducted through subgroup meta-analyses to examine potential sources of heterogeneity. Among the 26 studies employing the CIPS scale, substantial differences emerged according to sampling design (Table 2). Studies utilizing probabilistic sampling (n = 4; 2,872 participants) [25,26,28,39] reported a pooled prevalence of 36.5% (95% CI: 25.4%–48.4%; I² = 95%), while those with non-probabilistic sampling (n = 22; 6,238 participants) [17,19–21,23,24,27,29–36,38,41–43,45,46,49,50] yielded a higher prevalence of 57.5% (95% CI: 51.1%–63.7%; I² = 96%) (Table 2).

**Table 2:** Sensitivity analysis of IP prevalence in medical students according to CIPS.

Subgroup	Number of studies	Number of participants	Prevalence, % (95% CI)	I²
Sampling design				
Probabilistic	4	2,872	36.50 (25.35–48.42)	95%
Non-probabilistic	22	6,238	57.49 (51.13–63.72)	96%
Sex				
Female	17	3,838	50.54 (42.51–58.56)	96%
Male	17	3,081	47.13 (40.01–54.31)	93%
Academic stage				
Preclinical	8	1,178	48.87 (41.41–56.36)	84%
Clinical	10	1,762	46.82 (36.81–56.95)	94%
Region				
Africa	1	200	22.50 (17.26–28.77)	—
Asia	8	2,159	51.22 (44.95–57.47)	88%
North America	6	1,261	62.00 (45.94–76.83)	97%
Latin America	3	3,081	43.65 (28.45–59.50)	98%
Middle East	5	1,430	58.02 (37.34–77.34)	98%
Europe	3	979	61.49 (57.39–65.52)	41%
Country				
United Arab Emirates	1	399	89.47 (86.10–92.14)	—
Canada	1	277	75.81 (70.43–80.47)	—
United Kingdom	1	191	65.97 (58.95–72.21)	—
Romania	1	331	61.93 (56.59–66.99)	—
USA	5	984	59.00 (39.90–76.81)	97%
Sweden	1	457	58.42 (53.86–62.87)	—
Oman	1	276	52.17 (46.29–58.01)	—
Brazil	2	850	50.59 (45.06–56.11)	63%
Saudi Arabia	2	465	48.82 (36.96–60.75)	84%
India	3	815	48.45 (38.45–58.50)	87%
Thailand	2	500	46.60 (42.23–50.99)	0%
Malaysia	1	256	53.90 (47.79–59.91)	—
Bahrain	1	290	45.17 (39.54–50.93)	—
Peru	1	2,231	30.61 (28.66–32.50)	—
Pakistan	2	588	62.90 (56.85–68.74)	52%
Nigeria	1	200	22.50 (17.26–28.77)	—

Sex-stratified analysis revealed similar prevalences between groups. Among 17 studies reporting sex-specific data, the pooled prevalence was 50.5% (95% CI: 42.5%–58.6%; I² = 96%) in women (n = 3,838) and 47.1% (95% CI: 40.0%–54.3%; I² = 93%) in men (n = 3,081) [20,23–28,31–33,35,40–42,45,49]. Both estimates demonstrated substantial heterogeneity.

Analysis by academic stage showed comparable rates between training phases. Preclinical students (years 1–3, as operationalized for studies without explicit definitions) had a pooled prevalence of 48.9% (95% CI: 41.4%–56.4%; I² = 84%) across 8 studies (n = 1,178), while clinical students (year 4 onward) demonstrated 46.8% (95% CI: 36.8%–57.0%; I² = 94%) across 10 studies (n = 1,762) [20,21,23–26,31–33,35].

Regional analysis revealed considerable geographic variation. European studies (n = 3) reported the highest pooled prevalence at 61.5% (95% CI: 57.4%–65.5%; I² = 41%), followed by North America (n = 6) at 62.0% (95% CI: 45.9%–76.8%; I² = 97%) and the Middle East (n = 5) at 58.0% (95% CI: 37.3%–77.3%; I² = 98%). Asian studies (n = 8) showed 51.2% (95% CI: 45.0%–57.5%; I² = 88%), while Latin America (n = 3) reported 43.7% (95% CI: 28.5%–59.5%; I² = 98%). The single African study reported 22.5% (95% CI: 17.3%–28.8%) (Table 2).

Country-specific analyses demonstrated marked variation. The highest prevalence was observed in the UAE (89.5%; 95% CI: 86.1%–92.1%), followed by Canada (75.8%; 95% CI: 70.4%–80.5%) [42] and the United Kingdom (66.0%; 95% CI: 59.0%–72.2%) [29]. The USA, with five studies, showed a pooled prevalence of 59.0% (95% CI: 39.9%–76.8%; I² = 97%) [17,19,27,32,43]. Pakistan reported 62.9% (95% CI: 56.9%–68.7%; I² = 52%) across two studies [21,41]. Lower prevalences were observed in Thailand (46.6%; 95% CI: 42.2%–51.0%; I² = 0%) [24,38], Bahrain (45.2%; 95% CI: 39.5%–50.9%) [23], and Peru (30.6%; 95% CI: 28.7%–32.5%) [28]. Countries with multiple studies demonstrated heterogeneity ranging from 0% (Thailand) [24,38] to 97% (USA) [17,19,27,32,43] (Table 2).

### Meta-regression of IP prevalence (CIPS criteria) in medical students according to publication year

**Fig 4:**
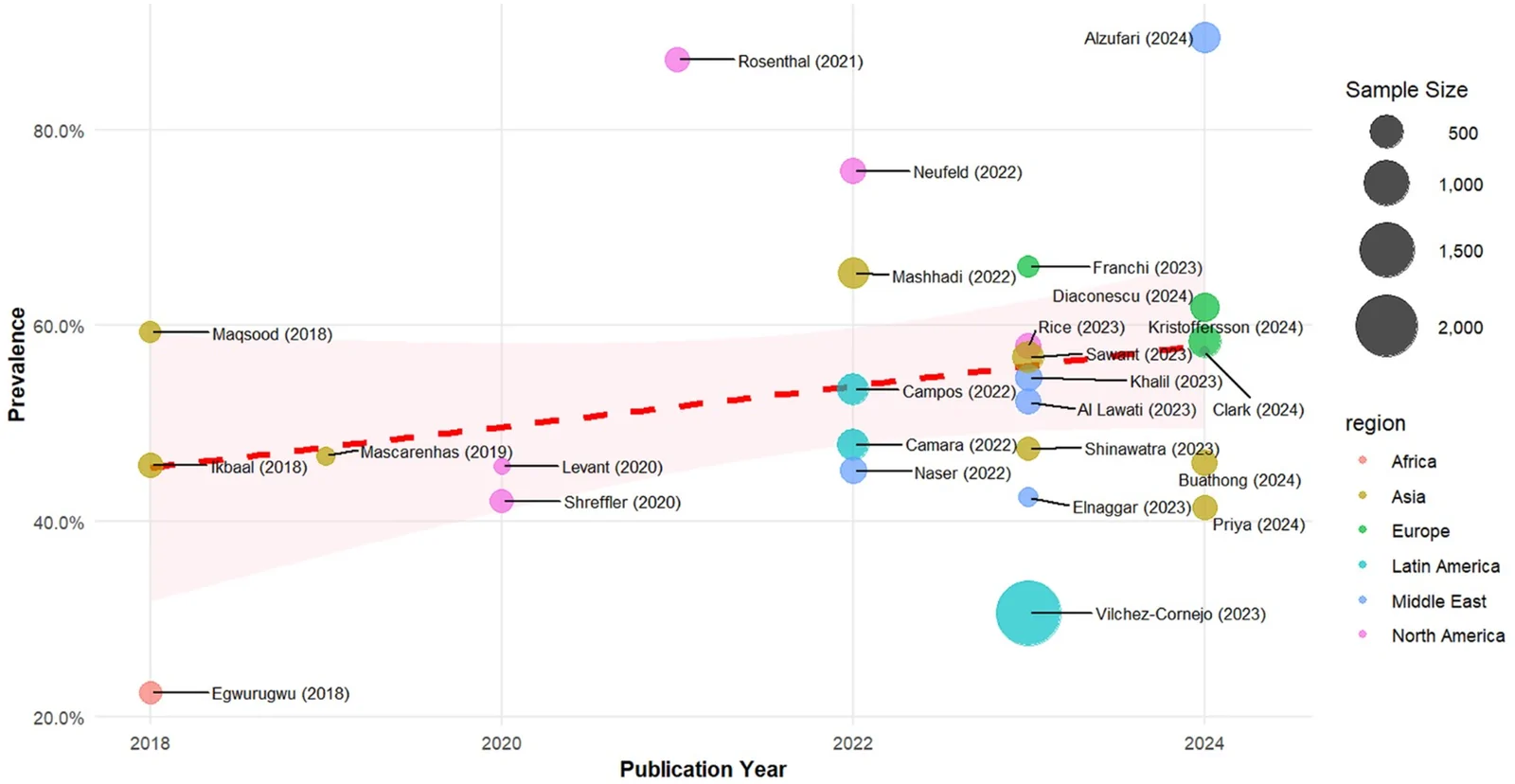
Meta-regression of the prevalence of IP (CIPS criteria) in medical students according to the year of publication. The red dashed line shows a temporal trend with a 95% confidence interval.

Meta-regression analysis was conducted to examine the relationship between publication year and IP prevalence among medical students (Fig 4). The model revealed a non-significant positive trend (β = 0.0221, SE = 0.0163, p = 0.1747), indicating no statistically significant temporal change in prevalence estimates from 2018 to 2024. The test of moderators (QM = 1.8419, df = 1, p = 0.1747) confirmed that publication year does not significantly explain the heterogeneity observed between studies. Residual heterogeneity remained substantial (I² = 97.08%, H² = 34.23, p < 0.0001), with the model accounting for only 3.31% of the heterogeneity (R² = 3.31%), suggesting that factors beyond temporal trends account for the vast majority of variation in prevalence estimates.

The bubble plot visualization revealed distinct patterns by geographic region and sample size. Studies conducted in North America (n = 6) demonstrated consistently elevated prevalences ranging from 42% to 87%, with persistence across the study period. Studies conducted in the Middle East similarly showed high prevalences (42% to 89%) throughout 2023–2024. Studies conducted in Asia displayed moderate variability, with prevalences between 41% and 65%. European studies, though limited in number (n = 3), reported prevalences from 58% to 62%. Latin American studies showed the widest range (31% to 53%), with the Vilchez-Cornejo (2023) study from Peru reporting both the lowest prevalence (30.6%) and the largest sample size (n = 2,231), while Brazilian studies reported higher rates (48% to 53%). African representation was limited to a single Nigerian study reporting 22.5% prevalence.

The regression line’s 95% confidence interval (shaded area) encompasses most data points, reflecting the high variability in prevalence estimates independent of publication year. The distribution of studies by sample size showed no clear relationship with prevalence estimates, as both large and small studies reported varied rates across the spectrum (Fig 4).

## Discussion

### Main findings

This systematic review and meta-analysis reveals that IP affects 54.2% (95% CI: 47.0%–61.3%) of medical students when assessed using the CIPS with conventional threshold-defined criteria (≥62 points). This prevalence, representing more than half of the medical student population experiencing moderate-to-severe impostor feelings, constitutes a relevant mental health and educational concern in medical training. However, this estimate derives predominantly from studies using non-probabilistic sampling. When restricted to studies employing probabilistic sampling methods, the prevalence decreases to 36.5% (95% CI: 25.4%–48.4%), though even this conservative estimate indicates that more than one-third of medical students experience threshold-defined IP.

Our meta-regression analysis revealed a non-significant positive trend (β = 0.0221, SE = 0.0163, p = 0.1747) from 2018 to 2024, suggesting that reported prevalence rates have not clearly declined over time. This temporal stability should be interpreted cautiously because meta-regression was ecological, underpowered for causal inference, and unable to account for institutional changes in student wellbeing initiatives. The model explained only 3.31% of the observed heterogeneity (R² = 3.31%), confirming that factors beyond temporal trends account for the vast majority of variation in prevalence estimates.

The distribution of IP showed remarkable consistency across demographic variables while revealing important geographic variations. Sex differences were minimal, with women showing 50.5% prevalence compared to 47.1% in men. This finding suggests that, within medical education contexts, intense evaluative and professional socialization pressures may reduce sex-based differences observed in some broader university or professional populations [4,5]. Similarly, prevalence rates between preclinical (48.9%) and clinical students (46.8%) were comparable, suggesting that increased clinical exposure does not necessarily alleviate impostor feelings. However, the definition of “preclinical” and “clinical” years varies across educational systems: in North American contexts, years 1–2 are typically preclinical and years 3–4 clinical, while in many other countries where students enter medical school directly from secondary education, medical training may span 5–7 years. Geographic variations were substantial, ranging from 22.5% in Africa to 62.0% in North America and 61.5% in Europe, with Asia demonstrating 51.2% prevalence.

### Methodological considerations and heterogeneity

The substantial heterogeneity observed in our meta-analysis (I² = 98%, τ² = 0.0338, p < 0.01) warrants careful examination because it fundamentally shapes the interpretation of our findings. While our analysis includes studies from 17 countries across six continental regions, we acknowledge that this represents only a fraction of global medical education systems. The term “global” in our context refers to multi-regional representation rather than universal coverage, a standard approach in international prevalence studies [10,11]. This heterogeneity should not be ignored; it reflects genuine and methodological variation across educational contexts, cultural settings, sampling strategies, and measurement practices.

We recognize that reporting a pooled global prevalence of 54.2% with very high heterogeneity generates legitimate methodological debate. Therefore, the pooled prevalence should not be interpreted as a single universal rate, but as the average of markedly variable estimates across educational, cultural, and methodological contexts. Even when considering only the most conservative estimates from probabilistic sampling studies (36.5%), more than one-third of medical students experience threshold-defined IP. Quantitative benchmarks are necessary to justify resource allocation, design targeted interventions, and monitor wellness programs, but these benchmarks must be interpreted alongside subgroup analyses and the observed range of estimates.

Medical education systems vary considerably worldwide. In the United States and Canada, students typically enter medical school after completing a prior undergraduate degree, beginning their medical training around age 22–24. In contrast, students in India, Pakistan, Peru, and many other countries enter medical programs directly from secondary school at younger ages, undertaking longer pre-licensure programs. These structural differences, combined with varying assessment philosophies and competitive pressures, may contribute meaningfully to the observed heterogeneity in IP prevalence. We did not conduct a subgroup meta-analysis by graduate-entry versus direct-entry medical education because this information was not consistently reported at the study or program level and country-level classification could have misclassified mixed-pathway systems.

We acknowledge that combining studies with different CIPS threshold approaches creates inherent methodological challenges. The inclusion of studies using CIPS thresholds of ≥61, ≥ 62, or ≥63 introduces variability in defining threshold-defined IP. Our decision to include these minor variations was guided by the fact that these cut-offs fall within closely related CIPS severity categories and are unlikely to represent meaningfully different constructs on a 20-item self-report scale [1,2].

The striking difference between studies using probabilistic sampling (36.5%; 95% CI: 25.4%–48.4%) versus non-probabilistic sampling (57.5%; 95% CI: 51.1%–63.7%) deserves particular attention. This 21-percentage point difference suggests that convenience sampling may substantially overestimate prevalence, possibly due to self-selection bias where students experiencing impostor feelings are more motivated to participate in relevant research [26,27,29,40]. However, even the lower estimate from probabilistic studies indicates that more than one-third of medical students experience threshold-defined IP, confirming this as a major educational challenge regardless of sampling methodology.

### Comparison with other studies and cultural factors

Previous systematic reviews in university and professional populations have reported IP prevalences ranging from 9% to 82%, with this wide variation largely attributable to different measurement instruments and threshold definitions [4]. Our focus on studies using the validated CIPS with comparable thresholds provides more interpretable estimates than the broader literature. Notably, our prevalence of 54.2% in medical students falls within the middle-to-upper range reported for practicing physicians and physicians in training (22% to 60%) in recent scoping reviews [6], suggesting that impostor feelings established during training may persist throughout medical careers.

The geographic variations we observed may reflect differences in educational philosophy, assessment culture, professional identity formation, and sampling methodology. North American medical education (n = 6 studies), with its competitive admissions, extensive standardized testing, and tradition of individual achievement, may create an environment where students frequently compare themselves to high-performing peers [16,17,19,20,27,32,43]. The similarly high rates in Europe (61.5%, n = 3 studies) may reflect competitive pressures across European medical schools [29,34,36]. Early clinical exposure in some curricula could also intensify impostor feelings as students confront the gap between developing competence and professional expectations.

Asian contexts showed substantial variation, with studies from India reporting 48.5% prevalence [30,33,49], Thailand 46.6% [24,38], Pakistan 62.9% [21,41], and Malaysia 53.9% [50]. These variations should not be reduced to simplistic cultural explanations. Cultural response patterns, institutional selectivity, assessment structures, social support, and sampling strategies may all contribute to the observed differences. In collectivist cultures, professional identity may be more closely tied to group membership and social roles rather than individual achievement, potentially buffering some forms of comparative self-evaluation, but this interpretation remains hypothesis-generating rather than conclusive [51].

The lower prevalence observed in Peru (30.6%) reported by Vilchez-Cornejo et al. [28], despite being the largest study in our review (n = 2,231) with probabilistic sampling, should be interpreted cautiously. It may reflect differences in sampling, institutional context, measurement implementation, cultural response patterns, or unmeasured educational factors. Further studies in Peru are needed to determine whether this finding is reproducible and to explore potential protective factors. Similarly, the single African study from Nigeria reporting 22.5% prevalence [39] does not allow continent-level inference; additional studies from African medical schools are required before drawing conclusions about regional prevalence or contextual protection.

### Implications for medical education reform

The persistence of high IP rates despite increased institutional attention to student wellbeing indicates that current approaches, primarily focused on individual resilience and stress management, may be insufficient [52,53]. Our temporal analysis showing stable prevalence rates suggests that awareness alone does not reduce IP prevalence. Similar patterns have been reported in physician mental health, where individual-level interventions remain common despite strong evidence that organizational and systemic factors contribute to psychological distress [53]. Understanding the scope and persistence of this phenomenon is prerequisite to designing effective interventions; resource allocation for wellness programs or curricular reforms requires empirical evidence of prevalence and impact [6,49].

The traditional “hidden curriculum” of medical education, with its emphasis on perfectionism, intellectual superiority, and emotional invulnerability, creates an environment where impostor feelings may flourish [17,20]. Students must navigate the profound identity transformation from layperson to physician while constantly being evaluated and compared to peers, a process central to professional identity formation in medical education [54]. The similarity in prevalence between preclinical and clinical years suggests that IP is not simply an adjustment reaction that resolves with experience but may reflect ongoing challenges in professional identity formation throughout medical training.

Because nearly all included studies were cross-sectional, reverse causation cannot be excluded. IP may contribute to psychological distress, but anxiety, depression, burnout, or low self-esteem may also intensify impostor feelings or influence how students respond to IP scales. This issue is especially relevant because medical students experience substantial burdens of depression, depressive symptoms, and suicidal ideation compared with many other student populations [55]. Longitudinal studies are needed to clarify temporal ordering. Current wellness programs often operate as add-on interventions that fail to address root causes. Rather than teaching stress management while maintaining unchanged competitive ranking systems, institutions should examine how their assessment practices and cultural norms contribute to IP [47,48].

The similar prevalence between male and female students should not obscure the need for targeted support for students with intersecting marginalized identities. International medical graduates, students from underrepresented minorities, first-generation college students, and those from lower socioeconomic backgrounds may face additional IP triggers related to cultural capital differences and systemic bias in medical education [27,48]. Future studies should report sex and gender variables precisely and avoid assuming that binary sex-stratified estimates capture the full range of identity-related vulnerability.

### Strengths and limitations

This systematic review possesses several methodological strengths: comprehensive search strategy across multiple databases without language restrictions, focus on validated instruments with established thresholds, large cumulative sample exceeding 9,100 students across 26 CIPS studies, systematic quality assessment using the Munn et al. tool, and extensive subgroup analyses. These strengths provide robust evidence for the scope of IP in medical education while allowing explicit assessment of methodological sources of variation.

Several limitations must be acknowledged. The extremely high heterogeneity indicates substantial unexplained variation among studies; therefore, the pooled prevalence should not be interpreted as a single universal rate, but as the average of markedly variable estimates across educational, cultural, and methodological contexts. The predominance of non-probabilistic sampling raises concerns about selection bias and limits generalizability. Nearly all included studies were cross-sectional, preventing causal inferences about factors contributing to IP or understanding individual trajectories over time. The uneven geographic distribution, with minimal African representation and absence from many regions, limits truly global conclusions. Self-report measures, while necessary for assessing subjective experiences, introduce potential biases including social desirability effects and cultural variations in psychological symptom reporting. The review protocol was not prospectively registered in PROSPERO, which may reduce transparency regarding deviations from planned methods. The exclusion of PsycINFO may have reduced sensitivity for psychology-focused studies despite use of multiple biomedical and multidisciplinary databases and manual reference checking. Restricting the search to studies published from 2000 onward may have excluded earlier reports, although this restriction was intended to prioritize contemporary medical education contexts and modern validated IP measurement. Inter-rater reliability was not formally quantified; although screening and data extraction were performed independently by two reviewers and disagreements were resolved by consensus or by a third reviewer, the absence of kappa statistics limits the assessment of reproducibility of the selection process. Finally, we could not conduct subgroup analyses according to medical program-entry structure, such as graduate-entry versus direct-entry medical education, because this information was not consistently reported at the study or program level.

### Future directions

Future research should prioritize methodological standardization to enable more reliable prevalence estimates. Researchers should adopt consistent measurement protocols using CIPS with standardized thresholds when possible, employ probabilistic sampling when feasible, report response rates transparently, and provide medical-student-specific numerators and denominators in mixed-population studies. This standardization will facilitate more robust meta-analyses and allow meaningful comparisons across contexts and time periods.

Initial institutional responses could include psychoeducational sessions that normalize IP, structured peer discussion, coaching or mentoring programs, and cognitive reframing strategies, all of which have emerging evidence in high-achieving or health-professional populations [52]. However, these approaches should be implemented with evaluation designs rather than assumed to be effective. Future intervention studies should also examine institutional-level strategies, including assessment practices, feedback culture, mentoring structures, and learning climate, because individual coping interventions alone may be insufficient when educational environments reinforce chronic comparison and perfectionism [53].

Medical education institutions should begin by measuring IP prevalence locally using validated instruments and by examining whether assessment practices, feedback cultures, and competitive ranking systems intensify impostor feelings. Longitudinal studies are needed to understand IP trajectories throughout medical training, examine which interventions show promise in different cultural contexts, and investigate the relationship between institutional practices and IP prevalence. Only through systematic investigation and evidence-based implementation can medical education begin to address this phenomenon among future physicians.

## Conclusions

In conclusion, IP is frequent among pre-licensure medical students, with CIPS-based prevalence estimates ranging from 36.5% in probabilistic studies to 54.2% in the overall synthesis. These estimates should be interpreted cautiously because between-study heterogeneity was substantial, sampling methods varied, and geographic coverage was uneven. Nevertheless, the consistency of elevated prevalence across diverse settings supports the need for standardized measurement, probabilistic sampling when feasible, longitudinal research, and rigorously evaluated interventions addressing both individual coping and institutional educational practices.

## Supporting information

S1 Table PRISMA 2020 checklist. (DOCX)

S2 Table Search strategy. (DOCX)

S3 Table Quality assessment of included studies using the Munn et al. critical appraisal tool for prevalence studies. (DOCX)

S1 Fig Funnel plot for assessment of publication bias in impostor phenomenon prevalence studies among medical students (Freeman-Tukey double arcsine transformation). (TIF)
