# Supplementary material for: Global prevalence of impostor phenomenon in medical students: A systematic review and meta-analysis
Source: PLoS One. 2026 Aug 26;21(8):e0351713. doi: 10.1371/journal.pone.0351713 (PMC13514014; doi:10.1371/journal.pone.0351713)
Supplement: Supplementary file 1 [file pone.0351713.s006.pdf]

Dear **Editor**,

Thank you very much for reviewing our article, " ***Global prevalence of impostor phenomenon in medical students: a systematic review and meta-analysis.***". Your suggestions and comments will be addressed below. Thank you for your valuable time and excellent review.

Editor's comments

1. Please upload a new copy of Figure 2 as the detail is not clear. Please follow the link for more information: http://journals.plos.org/plosone/s/figures.

   Our response: A clearer and enhanced version of Figure 2 has been uploaded. Additionally, the figure guidelines set by the journal have been reviewed and followed.

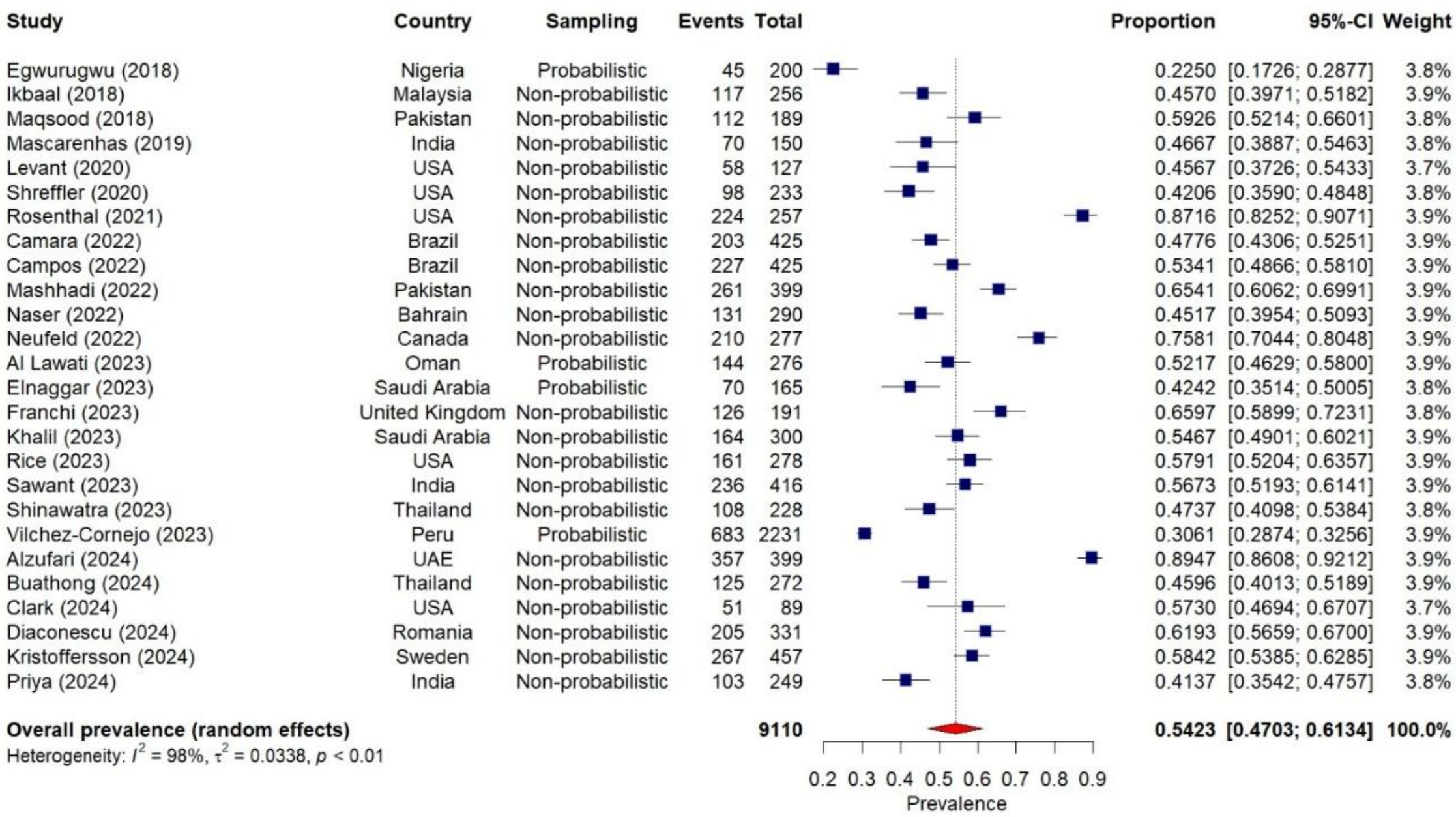

If you have any comments or recommendations, we are ready to respond.

Sincerely,

Mario J. Valladares-Garrido

Escuela de Medicina Humana, Universidad Señor de Sipán, Chiclayo, Peru; vgarrido@uss.edu.pe
