# Supplementary material for: Global prevalence of impostor phenomenon in medical students: A systematic review and meta-analysis
Source: PLoS One. 2026 Aug 26;21(8):e0351713. doi: 10.1371/journal.pone.0351713 (PMC13514014; doi:10.1371/journal.pone.0351713)
Supplement: Supplementary file 3 [file pone.0351713.s002.docx]

**Table S2.** Search strategy

| Search strategy in PUBMED | |
| --- | --- |
| #1 | "Impostor Syndrome"[tiab] OR "Imposter Phenomenon"[tiab] OR "Impostor phenomenon"[tiab] OR "Imposter Syndrome"[tiab] OR "síndrome del impostor"[tiab] OR "sindrome del impostor"[tiab] OR "impostor Syndrome"[tiab] OR "imposter phenomenon"[tiab] OR "fenómeno del impostor"[tiab] OR "fenomeno del impostor"[tiab] |
| #2 | "Students, Medical"[Mesh] OR "medical student"[tiab] OR "medical students"[tiab] OR "estudiante de medicina"[tiab] OR "estudiantes de medicina"[tiab] OR "undergraduate medical student"[tiab] OR "medical undergraduates"[tiab] |
| #3 | #1 AND #2 |
| Search strategy in SCOPUS | |
| #1 | "Impostor Syndrome" OR "Imposter Phenomenon" OR "Impostor phenomenon" OR "Imposter Syndrome" OR "síndrome del impostor" OR "sindrome del impostor" OR "impostor phenomenon" OR "imposter phenomenon" OR "fenómeno del impostor" OR "fenomeno del impostor" ) |
| #2 | "Students, Medical" OR "medical student" OR "medical students" OR "Estudiante de medicina" OR "estudiantes de medicina" OR "undergraduate medical student" OR "medical undergraduates" |
| #6 | TITLE-ABS-KEY ((#1 AND #2)) |
| Search strategy in Web of Science | |
| #1 | "Impostor Syndrome" OR "Imposter Phenomenon" OR "Impostor phenomenon" OR "Imposter Syndrome" OR "síndrome del impostor" OR "sindrome del impostor" OR "impostor phenomenon" OR "imposter phenomenon" OR "fenómeno del impostor" OR "fenomeno del impostor" |
| #2 | "Students, Medical" OR "medical student" OR "medical students" OR "estudiante de medicina" OR "estudiantes de medicina" OR "undergraduate medical student" OR "medical undergraduates" |
| #3 | TS=((#1 AND #2)) |
| Search strategy in EMBASE | |
| #1 | 'impostor Syndrome/exp OR 'imposter feelings' OR 'imposter phenomenon' OR 'imposter syndrome' OR 'imposterism' OR 'impostor experiences' OR 'impostor feelings' OR 'impostor phenomenon' OR 'impostor phenomenon' OR 'impostorism' |
| #2 | 'medical student'/exp OR 'med school student' OR 'med student' OR 'medical school student' OR 'medical student' OR 'medical students' OR 'student, medical' OR 'students, medical' |
| #3 | #1 AND #2 |
