# Supplementary material for: Global prevalence of impostor phenomenon in medical students: A systematic review and meta-analysis
Source: PLoS One. 2026 Aug 26;21(8):e0351713. doi: 10.1371/journal.pone.0351713 (PMC13514014; doi:10.1371/journal.pone.0351713)
Supplement: Supplementary file 4 [file pone.0351713.s003.docx]

**Table S3. Quality Assessment of Included Studies Using the Munn et al. Critical Appraisal Tool for Prevalence Studies**

Abbreviations: SF, sampling frame appropriate; PR, participants recruited appropriately; SS, sample size adequate; DS, subjects/setting described; DAC, data analysis coverage; VM, valid methods; SM, standard measurement; AS, adequate statistics; RR, response rate handled; Y, Yes; N, No/Unclear.

| Author (Year) | SF | PR | SS | DS | DAC | VM | SM | AS | RR | Total score | Risk level |
| --- | --- | --- | --- | --- | --- | --- | --- | --- | --- | --- | --- |
| Villwock (2016) | Y | N | Y | Y | Y | Y | Y | Y | N | 7 | Low risk |
| Qureshi (2017) | Y | N | Y | Y | Y | Y | Y | Y | N | 7 | Low risk |
| Ikbaal (2018) | Y | N | Y | Y | Y | Y | Y | Y | N | 7 | Low risk |
| Egwurugwu (2018) | Y | Y | Y | Y | Y | Y | Y | Y | N | 8 | Low risk |
| Maqsood (2018) | Y | N | Y | Y | Y | Y | Y | Y | N | 7 | Low risk |
| Holliday (2019) | Y | N | Y | Y | Y | Y | Y | Y | N | 7 | Low risk |
| Mascarenhas (2019) | Y | N | Y | Y | Y | Y | Y | Y | N | 7 | Low risk |
| Levant (2020a) | Y | N | Y | Y | Y | Y | Y | Y | N | 7 | Low risk |
| Shreffler (2020) | Y | N | Y | Y | Y | Y | Y | Y | N | 7 | Low risk |
| Levant (2020b) | Y | N | Y | Y | Y | Y | Y | Y | N | 7 | Low risk |
| Lee (2020) | Y | N | Y | Y | Y | Y | Y | Y | N | 7 | Low risk |
| Alsaleem (2021) | Y | Y | Y | Y | Y | Y | Y | Y | Y | 9 | Low risk |
| Rosenthal (2021) | Y | N | Y | Y | Y | Y | Y | Y | N | 7 | Low risk |
| Brennan-Wydra (2021) | Y | N | Y | Y | Y | Y | Y | Y | N | 7 | Low risk |
| Camara (2022) | Y | N | Y | Y | Y | Y | Y | Y | N | 7 | Low risk |
| Mashhadi (2022) | Y | N | Y | Y | Y | Y | Y | Y | N | 7 | Low risk |
| Shill-Russell (2022) | Y | N | Y | Y | Y | Y | Y | Y | N | 7 | Low risk |
| Naser (2022) | Y | N | Y | Y | Y | Y | Y | Y | N | 7 | Low risk |
| Campos (2022) | Y | N | Y | Y | Y | Y | Y | Y | N | 7 | Low risk |
| Neufeld (2022) | Y | N | Y | Y | Y | Y | Y | Y | N | 7 | Low risk |
| Shinawatra (2023) | Y | N | Y | Y | Y | Y | Y | Y | N | 7 | Low risk |
| Elnaggar (2023) | Y | Y | Y | Y | Y | Y | Y | Y | N | 8 | Low risk |
| Al Lawati (2023) | Y | Y | Y | Y | Y | Y | Y | Y | N | 8 | Low risk |
| Rice (2023) | Y | N | Y | Y | Y | Y | Y | Y | N | 7 | Low risk |
| Vilchez-Cornejo (2023) | Y | Y | Y | Y | Y | Y | Y | Y | Y | 9 | Low risk |
| Franchi (2023) | Y | N | Y | Y | Y | Y | Y | Y | N | 7 | Low risk |
| Sawant (2023) | Y | N | Y | Y | Y | Y | Y | Y | N | 7 | Low risk |
| Khalil (2023) | Y | N | Y | Y | Y | Y | Y | Y | N | 7 | Low risk |
| Clark (2024) | Y | N | Y | Y | Y | Y | Y | Y | N | 7 | Low risk |
| Priya (2024) | Y | N | Y | Y | Y | Y | Y | Y | N | 7 | Low risk |
| Diaconescu (2024) | Y | N | Y | Y | Y | Y | Y | Y | N | 7 | Low risk |
| Alzufari (2024) | Y | N | Y | Y | Y | Y | Y | Y | N | 7 | Low risk |
| Kristoffersson (2024) | Y | N | Y | Y | Y | Y | Y | Y | N | 7 | Low risk |
| Wrench (2024) | Y | N | Y | Y | Y | Y | Y | Y | N | 7 | Low risk |
| Buathong (2024) | Y | N | Y | Y | Y | Y | Y | Y | N | 7 | Low risk |

Note: Scores range from 0 to 9. Low risk = 7-9, moderate risk = 4-6, high risk = 0-3. A response rate was considered adequately handled only when the study reported sufficient information to assess non-response bias or explicitly addressed its potential influence on prevalence estimates.
