# Supplementary material for: Global prevalence of impostor phenomenon in medical students: A systematic review and meta-analysis
Source: PLoS One. 2026 Aug 26;21(8):e0351713. doi: 10.1371/journal.pone.0351713 (PMC13514014; doi:10.1371/journal.pone.0351713)
Supplement: Supplementary file 5 [file pone.0351713.s007.docx]

Thank you for the opportunity to review this work, which tackles a current issue impacting medical students and other intellectual groups. It is timely to examine the global prevalence of impostor syndrome among medical students, given their role as the future backbone of clinical care. However, I have some suggestions.

First, as a systematic review and meta-analysis, this provides the highest level of evidence, so all necessary steps must be taken to ensure thoroughness and transparency. The study should be registered on PROSPERO to prevent duplication and confirm its uniqueness. However, since the study has already been conducted, it is not possible to register with PROSPERO. Kindly take note of subsequent reviews.

To highlight the importance of the review, the introduction should note that impostor syndrome has been reported to have consequences in previous studies.

The researchers have done excellent work by adhering to the PRISMA Guideline, which ensures that the study is conducted in accordance with its principles. The latest PRISMA guideline is from 2020. Kindly indicate which version was used.
