# Supplementary material for: Global prevalence of impostor phenomenon in medical students: A systematic review and meta-analysis
Source: PLoS One. 2026 Aug 26;21(8):e0351713. doi: 10.1371/journal.pone.0351713 (PMC13514014; doi:10.1371/journal.pone.0351713)
Supplement: Supplementary file 6 [file pone.0351713.s008.docx]

**Response to Reviewers**

Dear Editor,

Thank you for the opportunity to revise our manuscript entitled “Global prevalence of impostor phenomenon in medical students: a systematic review and meta-analysis.” We carefully reviewed all editorial and reviewer comments and revised the manuscript and Supporting Information accordingly. All changes in the revised manuscript and revised supplementary files are highlighted in yellow.

**Editor**

**Reviewer/editor comment:** When submitting your revision, please ensure that your manuscript meets PLOS ONE’s style requirements, including those for file naming.

**Response:** Thank you for this instruction. We agree and have revised the manuscript and Supporting Information using PLOS ONE-compatible structure, clearer sectioning, and revised file names.

**Change made:** The revised files are provided as Word documents with clear file names. The manuscript also now includes title-page statements for funding, competing interests, and data availability.

**Reviewer/editor comment:** Thank you for stating the financial disclosure: “The publication fee (APC) was covered by Universidad Señor de Sipán (USS).” Please state what role the funders took in the study. If the funders had no role, please state: “The funders had no role in study design, data collection and analysis, decision to publish, or preparation of the manuscript.”

**Response:** Thank you for identifying this required statement. We agree and have added the funder role exactly as requested.

**Change made:** The title page now states: “Funding: The publication fee (APC) was covered by Universidad Señor de Sipán (USS). The funders had no role in study design, data collection and analysis, decision to publish, or preparation of the manuscript.”

**Reviewer #1**

**Reviewer/editor comment:** You switch between IP imposter phenomenon and IS imposter syndrome. An explanation is provided in the discussion. Would it be possible to use only IP? If not, consider moving this explanation up to the intro.

**Response:** Thank you for this observation. We agree that the terminology was inconsistent and that the conceptual explanation appeared too late in the manuscript.

**Change made:** We now use “IP” throughout the manuscript except when referring to terminology used by original studies, search strategies, or specific instruments. We moved the explanation to the Introduction and clarified that “impostor phenomenon” is preferred because it is not a formal psychiatric diagnosis and the term “syndrome” may be pathologizing.

**Reviewer/editor comment:** The section on study selection process is confusing. The 71 full text articles excluding 47 does not get to 35.

**Response:** Thank you for identifying this arithmetic inconsistency. We agree. The number of full-text exclusions was incorrect in the previous version.

**Change made:** We corrected the study selection paragraph and Figure 1. Seventy-one full-text articles were assessed; 36 were excluded; therefore, 35 studies were included in the qualitative synthesis. The full-text exclusion reasons are now reported as: failure to report IP prevalence data or sufficient data to calculate prevalence (n=9), use of nonvalidated instruments (n=10), and mixed populations without extractable medical-student-specific data (n=17).

**Reviewer/editor comment:** I am unclear why you included studies from 2016 forward but your metaregression only included 2018-2024?

**Response:** Thank you for noting this lack of clarity. We agree that the methods and results needed to specify the analytic subset used for meta-regression.

**Change made:** We clarified that the meta-regression was restricted to the primary CIPS-based quantitative synthesis. The earliest study eligible for that synthesis was published in 2018; therefore, the meta-regression covered 2018-2024, even though the qualitative synthesis included studies from 2016 onward.

**Reviewer/editor comment:** In the intro line 64 you state “reaching over 75% in certain educational contexts.” Adding a word or two to this sentence would help with clarity.

**Response:** Thank you for this suggestion. We agree that the phrase was vague.

**Change made:** The Introduction now specifies that these higher estimates occur in “competitive, high-stakes, and clinically supervised educational contexts.”

**Reviewer/editor comment:** I understand the importance of addressing well being also among physicians and leaders and note you highlighted the need for systems change.

**Response:** Thank you for this comment. We agree that the relevance of IP extends beyond individual student coping and should be interpreted in relation to educational systems.

**Change made:** We revised the Discussion and Future directions to frame institutional action more cautiously, emphasizing assessment practices, feedback culture, mentoring structures, and learning climate rather than making unsupported causal claims.

**Reviewer/editor comment:** I do not understand the wide heterogeneity documented in your study and will leave discussions about that to the statisticians.

**Response:** Thank you for raising this point. We agree that the high heterogeneity requires clearer interpretation for readers.

**Change made:** We revised the Methods and Discussion to explain that high I² is common in prevalence meta-analyses, that the pooled estimate should not be interpreted as a universal rate, and that subgroup analyses by sampling design, sex, academic stage, region, and country should guide interpretation.

**Reviewer #2**

**Reviewer/editor comment:** The impact of the study could be highlighted more effectively. I suggest revisiting the final sentence of the abstract and the final sentence of the introduction’s first paragraph.

**Response:** Thank you for this suggestion. We agree that the relevance of the study could be framed more clearly at key positions.

**Change made:** We revised the Abstract and Introduction to explain why medical students are vulnerable to IP and to emphasize that the study provides global CIPS-based prevalence estimates while requiring cautious interpretation because of heterogeneity.

**Reviewer/editor comment:** In the Introduction, it would be beneficial to briefly touch upon the conceptual nature of IS. Is it typically conceptualized as a stable, global personality trait or as a context-dependent state?

**Response:** Thank you for this important conceptual point. We agree that the manuscript needed this nuance.

**Change made:** We added text to the Introduction explaining that IP may show trait-like persistence in some individuals but may also be activated or intensified by high-stakes evaluative environments, professional identity transitions, and repeated comparison with high-performing peers.

**Reviewer/editor comment:** In the Methods section, you note that the work was not registered on a clinical trials registry. For systematic reviews, standard practice is registration in PROSPERO. Please state whether the review protocol was registered in PROSPERO. If not, acknowledge this as a limitation.

**Response:** Thank you for this correction. We agree that “clinical trial number: not applicable” did not adequately address systematic-review registration.

**Change made:** We replaced that statement with: “The review protocol was not prospectively registered in PROSPERO, and no registration number is available.” We also added the absence of prospective PROSPERO registration as a limitation.

**Reviewer/editor comment:** You acknowledge the exclusion of PsycINFO and provide a rationale. The argument would be strengthened if supported by evidence or data on database overlap or coverage.

**Response:** Thank you for this point. We agree that the previous wording was too confident. We did not want to imply that excluding PsycINFO was inconsequential.

**Change made:** We revised the Search strategy to state that PsycINFO was not searched because of limited institutional access, that this may have missed psychology-focused records, and that the limitation was mitigated by searching MEDLINE/PubMed, EMBASE, Scopus, Web of Science, and manually checking references. We added a methodological citation on database combinations and also acknowledged PsycINFO omission in the Limitations.

**Reviewer #3**

**Reviewer/editor comment:** This systematic review and meta-analysis is timely and addresses a pertinent issue. It was well conducted; however, the main challenge is the failure to register the protocol on PROSPERO, even though it is now too late to do so.

**Response:** Thank you for this comment. We agree. Because the review was not prospectively registered, we did not attempt retrospective registration or imply that the protocol had been prospectively registered.

**Change made:** We now explicitly state in Methods that the review protocol was not prospectively registered in PROSPERO and added this issue to the Limitations.

**Reviewer #4**

**Reviewer/editor comment:** General comment: consider reporting a prevalence rate for medical education programs based on graduate-level education versus direct entry from secondary education; consider reducing manuscript length; assess consistent use of IP and IS and run-on sentences.

**Response:** Thank you for these general comments. We partially agree. We agree that entry structure may be an important source of heterogeneity, but we did not conduct this subgroup analysis because program-entry structure was not consistently reported at the study level and country-level classification could misclassify mixed-pathway systems.

**Change made:** We added this issue to the Limitations. We also standardized terminology to IP, reorganized the Results using qualitative and quantitative synthesis headings, separated Future directions from Conclusions, and split or rewrote long sentences.

**Reviewer/editor comment:** Title: Based upon the conclusion, it seems that the authors are recommending both methodological changes as well as medical education reform. The title does not reflect these themes.

**Response:** Thank you for this observation. We partially agree. The title accurately reflects the design and primary objective of the manuscript, whereas the previous conclusion was broader than the evidence allowed.

**Change made:** We retained the title but narrowed the Conclusions and separated Future directions from Conclusions. The final conclusion now emphasizes prevalence, heterogeneity, standardized measurement, probabilistic sampling, longitudinal research, and rigorously evaluated interventions without overextending the title.

**Reviewer/editor comment:** Abstract, Introduction: consider adding one additional sentence to expand the background as to why medical students are an at-risk population.

**Response:** Thank you for this suggestion. We agree.

**Change made:** The Abstract and Introduction now specify that medical training combines repeated high-stakes assessment, competitive peer comparison, hierarchical clinical supervision, and rapid professional identity transition.

**Reviewer/editor comment:** Abstract, Objective: consider removing “undergraduate” as some included studies are conducted in the US where medical school is graduate education.

**Response:** Thank you for this important terminology correction. We agree.

**Change made:** We replaced “undergraduate medical students” with “pre-licensure medical students” in the Abstract, Methods, and relevant parts of the manuscript.

**Reviewer/editor comment:** Methodology: consider defining the CIPS scale to elaborate on why this IP measurement was chosen instead of alternative scales.

**Response:** Thank you for this suggestion. We agree that the analytic role of CIPS needed clarification.

**Change made:** We added that CIPS was treated as the primary instrument because it is the most frequently used IP measure in the included literature, uses a 20-item format, and provides conventional severity thresholds that allow more consistent prevalence synthesis. YIS and Leary scale studies are now summarized separately.

**Reviewer/editor comment:** Line 111: consider removing, repeated from line 100.

**Response:** Thank you for noting this repetition. We agree.

**Change made:** We revised the Selection criteria to avoid repeating the search date range and instead focused the section on eligibility criteria and synthesis grouping.

**Reviewer/editor comment:** Line 125: consider elaborating on examples of discrepancies.

**Response:** Thank you for this suggestion. We agree.

**Change made:** The Study selection process now specifies that discrepancies included disagreements about population eligibility, instrument validity, extractability of medical-student-specific prevalence, and compatibility of IP thresholds for quantitative synthesis.

**Reviewer/editor comment:** Results, Line 181: Should this be IP measurement?

**Response:** Thank you for catching this inconsistency. We agree.

**Change made:** We replaced “IS measurement” with “IP assessment” and standardized terminology throughout the manuscript.

**Reviewer/editor comment:** Results, Lines 184-187: Text does not match Figure 1. The text states that 35 studies met eligibility but Figure 1 states 24 studies in qualitative synthesis. Same for quantitative meta-analysis. Please correct.

**Response:** Thank you for identifying this inconsistency. We agree.

**Change made:** We corrected the study selection text and Figure 1. The revised PRISMA flow now reports 35 studies in qualitative synthesis, 26 studies in the primary CIPS meta-analysis, and 4 studies in the exploratory YIS synthesis.

**Reviewer/editor comment:** Results, Lines 205-207: consider separating into two sets of parentheses.

**Response:** Thank you for this formatting suggestion. We agree.

**Change made:** We revised the sentence describing study designs and sampling so that counts and percentages are clearer and not overloaded in a single parenthetical statement.

**Reviewer/editor comment:** Results, Line 216: reassess citation stating “unspecified in one study.”

**Response:** Thank you for this observation. We agree that the previous wording was unclear.

**Change made:** We revised the sentence to state that sex distribution was not reported in Clark (2024).

**Reviewer/editor comment:** Results, Line 245: consider elaborating or defining “inadequately handled response rates.”

**Response:** Thank you for this suggestion. We agree.

**Change made:** We now define this as response rates that were either not reported or not accompanied by an assessment of non-response bias, such as comparison between responders and non-responders or discussion of how low participation could affect prevalence estimates.

**Reviewer/editor comment:** Results, Line 351-352: consider changing to “studies in the Middle East...” “studies in Asia...” etc.

**Response:** Thank you for this wording suggestion. We agree.

**Change made:** We revised the meta-regression paragraph to use “studies conducted in North America,” “studies conducted in the Middle East,” and “studies conducted in Asia.”

**Reviewer/editor comment:** Discussion, Lines 376-380: lengthy sentence is unclear; may consider splitting into two sentences.

**Response:** Thank you for this comment. We agree.

**Change made:** We rewrote the relevant Discussion paragraph and split long sentences to improve readability.

**Reviewer/editor comment:** Discussion, Line 386: consider removing “clinically” given that IP is not a psychiatric condition.

**Response:** Thank you for this correction. We agree.

**Change made:** We replaced “clinically significant IP” and related phrasing with “threshold-defined IP” or “moderate-to-severe IP according to CIPS thresholds.”

**Reviewer/editor comment:** Discussion, Line 424: consider removing “policy makers, educators, and institutional leaders require” and changing to “quantitative benchmarks are necessary to justify resource allocation, design targeted interventions...”

**Response:** Thank you for this wording suggestion. We agree.

**Change made:** The sentence now states that quantitative benchmarks are necessary to justify resource allocation, design targeted interventions, and monitor wellness programs, while also emphasizing cautious interpretation due to heterogeneity.

**Reviewer/editor comment:** Discussion, Line 479: consider elaborating on unique features of Peruvian medical education.

**Response:** Thank you for this suggestion. We partially agree. We agree that the Peru finding requires discussion, but we avoided speculative claims about protective features without direct data.

**Change made:** We revised the paragraph to state that the lower prevalence observed in Peru may reflect sampling, institutional context, measurement implementation, cultural response patterns, or unmeasured educational factors. We also added that further studies in Peru are needed to determine reproducibility and explore potential protective factors.

**Reviewer/editor comment:** Conclusion: the conclusion does not match the title; the authors should consider narrowing the focus of the manuscript.

**Response:** Thank you for this comment. We agree that the previous conclusion was too broad.

**Change made:** We narrowed the final conclusion, separated Future directions from Conclusions, and framed educational reform as a future research and intervention priority rather than a direct causal inference from the meta-analysis.

**Reviewer/editor comment:** Table 1: Tables need footnotes to explain abbreviations used as well as scale for the risk of bias analysis.

**Response:** Thank you for this suggestion. We agree.

**Change made:** We added a Table 1 footnote defining CIPS, YIS, NR, IP, and the JBI/Munn risk-of-bias scoring range and categories.

**Reviewer/editor comment:** Table 1: In the Holliday (2019) study, was the data for medical students and dental medicine students separated for analysis?

**Response:** Thank you for raising this important point. We agree that this needed clarification.

**Change made:** We clarified in the Study selection section that some studies were retained for qualitative synthesis but were not included in the primary CIPS meta-analysis when pooling-compatible medical-student-specific prevalence data could not be extracted. Holliday was therefore not included in the primary CIPS quantitative synthesis.

**Reviewer/editor comment:** Figures: All figures are blurry and need high-resolution images.

**Response:** Thank you for identifying this presentation issue. We agree.

**Change made:** We corrected the PRISMA flow diagram and provide an updated high-resolution Figure 1. Figure captions were also corrected to use IP consistently. The figure files are provided separately for replacement during resubmission.

**Reviewer #5**

**Reviewer/editor comment:** Summary: The paper estimates the global prevalence of IP in undergraduate medical students and analyzes differences by sex, academic level, and geographic region. Minor additions and adjustments could strengthen the manuscript.

**Response:** Thank you for the positive assessment and constructive recommendations. We agree with the need for clarification and have revised the manuscript accordingly.

**Change made:** We changed “undergraduate” to “pre-licensure,” clarified CIPS and YIS syntheses, standardized terminology, added PROSPERO and PsycINFO limitations, revised PRISMA counts, updated Table 2, and expanded Table S3 to all 35 studies.

**Reviewer/editor comment:** Introduction 1: Line 57-59: state what population the outcomes/ref 3 are in — all professionals or students?

**Response:** Thank you for this point. We agree that the population context was unclear.

**Change made:** We revised the Introduction to state that these consequences have been described in student and health-professional populations.

**Reviewer/editor comment:** Introduction 2: Line 65-66: if ref [5] is a meta-analysis on IP in medical students, state clearly how your study updates/expands/adds value.

**Response:** Thank you for this observation. We agree that the relationship with previous reviews needed clarification.

**Change made:** We clarified that prior reviews have examined physicians and physicians in training more broadly, whereas the present study is a dedicated global meta-analysis focused on pre-licensure medical students and explores instruments, sampling strategies, academic stages, and regions.

**Reviewer/editor comment:** Introduction 3: Consider adding a sentence contextualising why medical students would be especially vulnerable to IP.

**Response:** Thank you for this suggestion. We agree.

**Change made:** We added text explaining that repeated high-stakes assessment, competitive peer comparison, hierarchical clinical supervision, early patient-care responsibility, and professional identity formation may make medical students particularly vulnerable.

**Reviewer/editor comment:** Introduction 4: Change to “This systematic review and meta-analysis.”

**Response:** Thank you. We agree.

**Change made:** We replaced “This systematic review (SR)” with “This systematic review and meta-analysis.”

**Reviewer/editor comment:** Method 1: State if your review was registered on PROSPERO or not.

**Response:** Thank you. We agree.

**Change made:** We now state that the review protocol was not prospectively registered in PROSPERO and added this as a limitation.

**Reviewer/editor comment:** Method 2: Add a reason for why your start date for the search was January 1 2000 and not from the beginning of databases, and discuss how this may have impacted results.

**Response:** Thank you for this point. We agree.

**Change made:** We added that the year 2000 was selected to focus on contemporary medical education contexts and studies using modern validated IP instruments. We also added to the Limitations that this restriction may have excluded earlier reports.

**Reviewer/editor comment:** Method 3: Mixed populations were accepted if data specific to medical students could be extracted. Was this for any proportion of medical students?

**Response:** Thank you for requesting clarification. We agree that this eligibility rule needed to be more explicit.

**Change made:** We clarified that no minimum proportion of medical students was required when medical-student-specific prevalence data were available. We also clarified that quantitative synthesis required extractable numerator and denominator for medical students.

**Reviewer/editor comment:** Method 4: Include metrics for inter-rater reliability between screeners, e.g., kappa.

**Response:** Thank you for this recommendation. We agree that kappa would be useful, but individual pre-consensus decisions were not retained in a format suitable for reliable estimation. We therefore did not calculate or invent an inter-rater statistic.

**Change made:** We added this to the Methods and Limitations: formal inter-rater reliability was not calculated because individual pre-consensus screening decisions were not retained in a suitable format.

**Reviewer/editor comment:** Method 5: Line 130: remove capitalisation on “Data.”

**Response:** Thank you. We agree.

**Change made:** We changed “Data extraction” to “data extraction” in the sentence.

**Reviewer/editor comment:** Method 6: Line 144: add initials for quality reviewers.

**Response:** Thank you. We agree.

**Change made:** We added reviewer initials for risk-of-bias assessment: VJV-P and FEZ-M, with MJV-G resolving disagreements when needed.

**Reviewer/editor comment:** Method 7: Line 154-156: add a reference for the theoretical expectation that true prevalence varies across medical education contexts.

**Response:** Thank you for this suggestion. We agree.

**Change made:** We added a methodological reference supporting random-effects proportional meta-analysis in the presence of expected contextual variability.

**Reviewer/editor comment:** Method 8: Line 160-162: add a reference for interpreting high I² values in global prevalence studies.

**Response:** Thank you. We agree.

**Change made:** We added references explaining that high I² values are common in prevalence meta-analyses and should be interpreted cautiously rather than as an automatic reason not to synthesize.

**Reviewer/editor comment:** Method 9: The terms “sex” and “gender” are used interchangeably. Please ensure consistency.

**Response:** Thank you for this important terminology correction. We agree.

**Change made:** We standardized the manuscript to “sex” when referring to variables reported as male/female in the original studies and added “sex as reported by the original studies” in Data extraction.

**Reviewer/editor comment:** Method 10: Sensitivity analyses were mentioned in the ROB section but not described in Statistical analysis. Clarify if planned but not conducted because all studies were low risk.

**Response:** Thank you. We agree that this was unclear.

**Change made:** We clarified that risk-of-bias-based sensitivity analyses were planned but not performed because all included studies were classified as low risk. We also clarified that subgroup analyses were used to explore heterogeneity.

**Reviewer/editor comment:** Method 11: Make clearer that one meta-analysis was done with CIPS studies and another subgroup/exploratory analysis with YIS studies; state whether planned or post-hoc and which findings are emphasized.

**Response:** Thank you for this suggestion. We agree.

**Change made:** We clarified that the CIPS meta-analysis was the primary quantitative synthesis, while the YIS synthesis was exploratory because YIS differs from CIPS in item number, scoring, and threshold definition. Results now repeatedly identify CIPS findings as the primary pooled estimates.

**Reviewer/editor comment:** Method 12: Define preclinical and clinical with years in the Methods because this varies across countries.

**Response:** Thank you. We agree.

**Change made:** We added that academic stage was operationalized according to each study’s definitions; when not explicitly defined, years 1-3 were classified as preclinical and year 4 onward as clinical, recognizing variation across educational systems.

**Reviewer/editor comment:** Results 1: Line 180: “non-corresponding populations of undergraduate medical students” needs clearer definition.

**Response:** Thank you. We agree.

**Change made:** We changed this to “studies that did not involve pre-licensure medical students.”

**Reviewer/editor comment:** Results 2: Line 187: six studies using YIS were excluded from the meta-analysis; cite the studies and clarify that YIS studies were examined separately.

**Response:** Thank you for identifying this confusing wording. We agree.

**Change made:** We corrected the text to state that five studies used YIS. Four YIS studies with comparable thresholds were included in an exploratory synthesis, while Qureshi et al. was summarized qualitatively because its outcome definition was not directly comparable.

**Reviewer/editor comment:** Results 3: Move the description of the nine Munn tool criteria from Results to Methods.

**Response:** Thank you. We agree.

**Change made:** We moved the nine JBI/Munn criteria to the Methods under Risk of bias assessment.

**Reviewer/editor comment:** Results 4: Add headings for “Qualitative results” and “Quantitative results.”

**Response:** Thank you for this recommendation. We agree.

**Change made:** We reorganized Results using clearer headings: Study selection; Qualitative synthesis; Risk of bias assessment; Quantitative synthesis; Exploratory YIS synthesis; Subgroup and sensitivity analyses; Meta-regression; and Reporting bias assessment.

**Reviewer/editor comment:** Results 5: “All studies (100%) met criteria for using validated instruments (CIPS)” should include N and account for YIS studies.

**Response:** Thank you. We agree.

**Change made:** We revised this sentence to state that all included studies used validated IP instruments, including CIPS, YIS, or the Leary Impostor Scale. Risk-of-bias assessment now covers all 35 studies rather than only 26 CIPS studies.

**Reviewer/editor comment:** Results 6: “Minor variations in cut-off points (≥61 or ≥63) were considered clinically equivalent” needs a reference or precedent.

**Response:** Thank you. We agree that the word “clinically” was inappropriate and that the rationale needed clarification.

**Change made:** We changed the phrase to “treated as comparable” and explained that these cut-offs fall within closely related CIPS severity categories and do not represent meaningfully different constructs on the 20-item scale.

**Reviewer/editor comment:** Results 7: In the YIS subanalysis, state why only 4 studies were included and cite the excluded studies and why.

**Response:** Thank you. We agree.

**Change made:** We clarified that four YIS studies had comparable dichotomous thresholds and that Qureshi et al. was summarized qualitatively because its outcome definition was not directly comparable with the threshold used in the exploratory YIS synthesis.

**Reviewer/editor comment:** Discussion 1: Explanation of why the term phenomenon rather than syndrome is used should be moved to the Introduction.

**Response:** Thank you. We agree.

**Change made:** We moved the terminology explanation to the Introduction and standardized the manuscript to IP.

**Reviewer/editor comment:** Discussion 2: Did you mean “clinically significant IP” or clinical levels of IP? Provide a reference if claiming clinical significance.

**Response:** Thank you. We agree that this wording was problematic because IP is not a diagnosable psychiatric condition.

**Change made:** We replaced this terminology with “threshold-defined IP” and “moderate-to-severe IP according to CIPS thresholds.”

**Reviewer/editor comment:** Discussion 3: Good depth of consideration of implications of high heterogeneity on results and confidence.

**Response:** Thank you for this positive comment.

**Change made:** We further strengthened this section by emphasizing that the pooled estimate should not be interpreted as a universal rate and by adding methodological references on prevalence meta-analysis heterogeneity.

**Reviewer/editor comment:** Discussion 4: Add that further research needs to be conducted in Peru and Africa to verify findings and in Peru to explore potentially protective factors.

**Response:** Thank you. We agree, with the caveat that protective factors should not be inferred without direct evidence.

**Change made:** We revised the Peru and Africa paragraph to state that further studies are needed in Peru to determine reproducibility and explore potential protective factors, and that additional African studies are required before continent-level inference.

**Reviewer/editor comment:** Discussion 5: Add citations for current individual-level wellbeing approaches and increased institutional awareness; similar patterns are observed in mental health despite increased awareness, with individual-level interventions despite systemic issues, e.g., Petrie et al. 2025.

**Response:** Thank you for this important point. We agree.

**Change made:** We added citations to intervention literature and incorporated Petrie et al. 2025 to support the statement that physician mental-health interventions often remain individual-level despite organizational contributors.

**Reviewer/editor comment:** Discussion 6: Add a reference in the professional identity formation discussion.

**Response:** Thank you. We agree.

**Change made:** We added a professional identity formation reference to the Discussion.

**Reviewer/editor comment:** Discussion 7: Add a sentence or two highlighting potential reverse causation that cannot be addressed by cross-sectional designs, especially given high rates of mental ill-health among medical students.

**Response:** Thank you. We agree.

**Change made:** We added a paragraph explaining that IP may contribute to distress, but anxiety, depression, burnout, or low self-esteem may also intensify impostor feelings or influence responses to IP scales. We also added a reference on depression and suicidal ideation among medical students.

**Reviewer/editor comment:** Discussion 8: Add a reference for “contrasting with wider gender gaps in other populations” and clarify which populations.

**Response:** Thank you. We agree.

**Change made:** We revised the statement to refer more cautiously to broader university and professional populations and cited existing IP literature. We also added a recommendation that future studies report sex and gender variables precisely.

**Reviewer/editor comment:** Discussion 9: High heterogeneity means pooled rates should be taken with considerable caution; include this in conclusion and abstract.

**Response:** Thank you. We agree.

**Change made:** We added cautionary language to the Abstract, Discussion, Limitations, and final Conclusions, stating that pooled estimates should not be interpreted as a single universal rate.

**Reviewer/editor comment:** Discussion 10: Separate Future directions/recommendations from Conclusion into separate paragraphs and headings.

**Response:** Thank you. We agree.

**Change made:** We separated the final section into “Future directions” and “Conclusions.”

**Reviewer/editor comment:** Discussion 11: In recommendations, cite evidence-based strategies or interventions for IP in medical students or other populations.

**Response:** Thank you. We agree.

**Change made:** We revised the Future directions section to include psychoeducation, structured peer discussion, coaching or mentoring, and cognitive reframing strategies as approaches with emerging evidence, while emphasizing that these should be implemented with evaluation designs rather than assumed to be effective.

Sincerely,

Mario J. Valladares-Garrido

Escuela de Medicina Humana, Universidad Señor de Sipán, Chiclayo, Peru; vgarrido@uss.edu.pe
