# Supplementary material for: Global prevalence of impostor phenomenon in medical students: A systematic review and meta-analysis
Source: PLoS One. 2026 Aug 26;21(8):e0351713. doi: 10.1371/journal.pone.0351713 (PMC13514014; doi:10.1371/journal.pone.0351713)
Supplement: Peer review history

# Author comment: Author response to Decision Letter 0

**DOI:** 10.1371/journal.pone.0351713.r001

11 Dec 2025

Attachment Submitted filename: Point-by-point response.pdf

# Peer review: Decision Letter 0

**DOI:** 10.1371/journal.pone.0351713.r002

**Authors:** Miquel Vall-llosera Camps

30 Jun 2026

PONE-D-25-65587

Global prevalence of impostor phenomenon in medical students: a systematic review and meta-analysis

PLOS One

Dear Dr. Valladares-Garrido,

Thank you for submitting your manuscript to PLOS ONE. After careful consideration, we feel that it has merit but does not fully meet PLOS ONE’s publication criteria as it currently stands. Therefore, we invite you to submit a revised version of the manuscript that addresses the points raised during the review process.

I would like to sincerely apologise for the delay you have incurred with your submission. It has been exceptionally difficult to secure reviewers to evaluate your study. We have now received five completed reviews; the comments are available below. The reviewers have raised significant scientific concerns about the study that need to be addressed in a revision.

Please revise the manuscript to address all the reviewer's comments in a point-by-point response in order to ensure it is meeting the journal's publication criteria. Please note that the revised manuscript will need to undergo further review, we thus cannot at this point anticipate the outcome of the evaluation process.

Please submit your revised manuscript by Aug 14 2026 11:59PM. If you will need more time than this to complete your revisions, please reply to this message or contact the journal office at plosone@plos.org. When you're ready to submit your revision, log on to https://www.editorialmanager.com/pone/ and select the 'Submissions Needing Revision' folder to locate your manuscript file.

Please include the following items when submitting your revised manuscript:

- A letter that responds to each point raised by the academic editor and reviewer(s). You should upload this letter as a separate file labeled 'Response to Reviewers'.
- A marked-up copy of your manuscript that highlights changes made to the original version. You should upload this as a separate file labeled 'Revised Manuscript with Track Changes'.
- An unmarked version of your revised paper without tracked changes. You should upload this as a separate file labeled 'Manuscript'.

If you would like to make changes to your financial disclosure, please include your updated statement in your cover letter. Guidelines for resubmitting your figure files are available below the reviewer comments at the end of this letter.

If applicable, we recommend that you deposit your laboratory protocols in protocols.io to enhance the reproducibility of your results. Protocols.io assigns your protocol its own identifier (DOI) so that it can be cited independently in the future. For instructions see: https://journals.plos.org/plosone/s/submission-guidelines#loc-laboratory-protocols. Additionally, PLOS ONE offers an option for publishing peer-reviewed Lab Protocol articles, which describe protocols hosted on protocols.io. Read more information on sharing protocols at https://plos.org/protocols?utm_medium=editorial-email&utm_source=authorletters&utm_campaign=protocols.

As the corresponding author, your ORCID iD is verified in the submission system and will appear in the published article. PLOS supports the use of ORCID, and we encourage all coauthors to register for an ORCID iD and use it as well. Please encourage your coauthors to verify their ORCID iD within the submission system before final acceptance, as unverified ORCID iDs will not appear in the published article. *Only*  the individual author can complete the verification step; PLOS staff *cannot*  verify ORCID iDs on behalf of authors.

We look forward to receiving your revised manuscript.

Kind regards,

Miquel Vall-llosera Camps

Senior Staff Editor

PLOS One

Journal requirements:

When submitting your revision, we need you to address these additional requirements.

1. Please ensure that your manuscript meets PLOS ONE's style requirements, including those for file naming. The PLOS ONE style templates can be found at

https://journals.plos.org/plosone/s/file?id=wjVg/PLOSOne_formatting_sample_main_body.pdf and

https://journals.plos.org/plosone/s/file?id=ba62/PLOSOne_formatting_sample_title_authors_affiliations.pdf

2. Thank you for stating the following financial disclosure:

“The publication fee (APC) was covered by Universidad Señor de Sipán (USS).”

Please state what role the funders took in the study.  If the funders had no role, please state: "The funders had no role in study design, data collection and analysis, decision to publish, or preparation of the manuscript."

If this statement is not correct you must amend it as needed.

Please include this amended Role of Funder statement in your cover letter; we will change the online submission form on your behalf.

If the reviewer comments include a recommendation to cite specific previously published works, please review and evaluate these publications to determine whether they are relevant and should be cited. There is no requirement to cite these works unless the editor has indicated otherwise.

[Note: HTML markup is below. Please do not edit.]

Reviewers' comments:

Reviewer's Responses to Questions

**Comments to the Author**

1. Is the manuscript technically sound, and do the data support the conclusions?

The manuscript must describe a technically sound piece of scientific research with data that supports the conclusions. Experiments must have been conducted rigorously, with appropriate controls, replication, and sample sizes. The conclusions must be drawn appropriately based on the data presented.

Reviewer #1: Partly

Reviewer #2: Yes

Reviewer #3: Yes

Reviewer #4: Partly

Reviewer #5: Yes

**********

2. Has the statistical analysis been performed appropriately and rigorously?

Reviewer #1: I Don't Know

Reviewer #2: Yes

Reviewer #3: Yes

Reviewer #4: Yes

Reviewer #5: Yes

**********

3. Have the authors made all data underlying the findings in their manuscript fully available?

The PLOS Data policy requires authors to make all data underlying the findings described in their manuscript fully available without restriction, with rare exception (please refer to the Data Availability Statement in the manuscript PDF file). The data should be provided as part of the manuscript or its supporting information, or deposited to a public repository. For example, in addition to summary statistics, the data points behind means, medians and variance measures should be available. If there are restrictions on publicly sharing data—e.g. participant privacy or use of data from a third party—those must be specified.

Reviewer #1: Yes

Reviewer #2: Yes

Reviewer #3: Yes

Reviewer #4: Yes

Reviewer #5: Yes

**********

4. Is the manuscript presented in an intelligible fashion and written in standard English?

PLOS ONE does not copyedit accepted manuscripts, so the language in submitted articles must be clear, correct, and unambiguous. Any typographical or grammatical errors should be corrected at revision, so please note any specific errors here.

Reviewer #1: Yes

Reviewer #2: Yes

Reviewer #3: Yes

Reviewer #4: Yes

Reviewer #5: Yes

**********

5. Review Comments to the Author

Please use the space provided to explain your answers to the questions above. You may also include additional comments for the author, including concerns about dual publication, research ethics, or publication ethics. (Please upload your review as an attachment if it exceeds 20,000 characters)

Reviewer #1: The manuscript is well written, address a relevant issue in medicl training and practice,with significant opportunites

for further rerserach and potential remedy.

Your analysis is appropriately placed within the body of literature ( It is a systemic review). Your methods are described in sufficient detail to enable reproducibility of the study

Below are some questions for clarity and potential edits to consider

1. You switch between IP imposter phenomenon and IS imposter syndrome. An explanation is provided in the discussion . Would it be possible t use only IP ? If not ( ? validated tool /litearure use IS ) consider moving this explanation up to the intro

2. The section on study selection process the written section is confusing . The 71 full text articles excluding 47 does not get to 35 ??

3) I am unclear why you included studies from 2016 forward but your metaregression only included 2018-2024?

4) In the intro line 64 you state ...... reaching over 75 % in certain educational contexts. Adding a word or two to this sentence would help with clariity ( what contexts ?/)

5) I understand the importance of addressing well being also among physicains and leaders and note you highlighted the need for systems change

6 ) I dont understand the wide heterogeneity documented in your study and will leave discussions about that to the statisticians

Good luck

Reviewer #2: I would like to commend you on this timely systematic review. The study appears to be well conducted. The manuscript is generally well-written, with a logical flow that makes it easy to follow, and the most relevant details needed to understand the analysis and its implications are present in the text. Your conclusions are valuable for understanding differences in the prevalence of Impostor Syndrome (IS) and its potential causes and implications across different regions and educational settings.

My suggestions below are intended to further strengthen the manuscript.

1. This is a high-quality study, and its impact could be highlighted even more effectively. I suggest revisiting the final sentence of the abstract and the final sentence of the introduction's first paragraph. These key positions offer an opportunity to frame the study’s main takeaway or its significance more powerfully, ensuring the reader immediately grasps the importance of your work.

2. In the Introduction, it would be beneficial to briefly touch upon the conceptual nature of IS. Is it typically conceptualized as a stable, global personality trait affecting a person’s general self-perception, or is it more of a context-dependent state, specific to certain skills or environments (e.g., clinical skills vs. academic knowledge)? A sentence or two clarifying this distinction would add valuable nuance for the reader.

3. In the Methods section, you note that the work was not registered on a clinical trials registry. However, for systematic reviews, the standard practice is registration in a dedicated repository like PROSPERO. Could you please state whether the review protocol was registered in PROSPERO? If so, please provide the registration number. If not, I suggest briefly acknowledging this as a limitation, in line with PRISMA guidelines.

4. You acknowledge the exclusion of the PsycINFO database and provide a rationale. While your reasoning is plausible, the argument would be significantly strengthened if it were supported by evidence. Could you provide a reference or data (e.g., from a study on database overlap or coverage in medical education research) to substantiate the claim that the selected databases likely captured most relevant literature? This would add an extra layer of methodological rigor to your search strategy.

Reviewer #3: This systematic review and meta-analysis is timely and addresses a pertinent issue. It was well conducted; however, the main challenge is the failure to register the protocol on Prospero, even though it is now too late to do so.

Reviewer #4: General comments to the author:

The authors conducted a systematic review and meta-analysis reviewing the global prevalence of the imposter phenomenon amongst medical students. Overall, the authors should consider reporting a prevalence rate for medical educations programs based on the discrepancy between medical education being graduate-level education in some countries versus directly from secondary education. The authors should also consider reducing the length of the manuscript since much of the text focuses on statistical methods, rather than the focus of the paper, which is the global prevalence of IP in medical students. There is only one page that discusses the study in context of other studies, which seems minimal for a 30-page manuscript. Additionally, the authors should assess the manuscript for consistent use of IP and IS and run-on sentences.

Specific Comments:

• Title: Based upon the conclusion, it seems that the authors are recommending both methodological changes as well as medical education reform. The title does not reflect these themes.

• Abstract:

o Introduction: consider adding one additional sentence to expand the background as to why medical students are an at-risk population

o Objective: consider removing “undergraduate” as some of the studies included are conducted in the US where medical school is graduate education

• Main text:

o Methodology:

� Consider defining the CIPS scale to elaborate on why this IP measurement was chosen instead of the alternative scales

� Line 111: consider removing, repeated from line 100

� Line 125: consider elaborating on examples of discrepancies

o Results:

� Line 181: Should this be IP measurement?

� Line 184-187: Text does not match Figure 1. In the text, the authors state that 35 studies met all eligibility but in Figure 1, it states that there are 24 studies that were included in qualitative synthesis. Same with the number of studies included in the quantitative meta-analysis. Please correct.

� Line 205-207: consider separating into two sets of parentheses

� Line 216: reassess citation stating “unspecified in one study”

� Line 245: consider elaborating or defining “inadequately handled response rates”

� Line 351-352: consider changing to “studies in the Middle East...” “studies in Asia...” etc.

o Discussion:

� Line 376-380: Lengthy sentence is unclear; may consider splitting into two sentences.

� Line 386: consider removing “clinically” given that IP is not a psychiatric condition as described previously

� Line 424: consider removing “policy makers, educators, and institutional leaders require” and changing to “quantitative benchmarks are necessary to justify resource allocation, design targeted interventions...”

� Line 479: consider elaborating on unique features of Peruvian medical education

o Conclusion:

� As stated above, the conclusion does not match the title of the manuscript; the authors should consider narrowing the focus of the manuscript

o Table 1:

� Tables need footnotes to explain abbreviations used as well as scale for the risk of bias analysis.

� In the Holliday (2019) study: was the data for medical students and dental medicine students separated for analysis?

o Figures:

All figures are blurry – need high resolution images

Reviewer #5: "Global prevalence of impostor phenomenon in medical students: a systematic review and meta-analysis"

SUMMARY:

This paper describes the results of a systematic review and meta-analysis to estimate the global prevalence of IP in undergraduate medical students and analyze differences by sex, academic level, and geographic region. This manuscript addresses an important topic and is well-written, comprehensive in analytic approach and in discussion. The study method and analyses were appropriate for the aims and for the outcome in consideration. A number of minor additions and adjustments could be included to strengthen the manuscript prior to publication, but overall, a solid paper with comprehensive, thoughtful discussion.

INTRODUCTION

1. LINE 57-59, PG 11: “The consequences of this phenomenon are significant and include increased risk of anxiety, depression, and burnout, contributing to the deterioration of psychological well being, professional exhaustion, and even increased risk of suicidal ideation [3].”

- State what population these outcomes/ref 3 is in – is it all professionals? Or students?

2. Line 65-66, pg 11: you say here there is limited synthesis related ot med students – but is ref [5] a meta-analysis on IP in medical students? If so, state clearly how your study updates / expands / adds value to this previous ref 5 – and/or if there are other reviews in this population, how yours differs?

3. Consider adding a sentence contextualising WHY med students would be especially vulnerable to IP? You mention challenges they face in line 69 – maybe give us a bit more context of these so we can understand the context for IP arising and its role in their education.

4. Line 70, pg 11: Change to: “This systematic review (sr) and META-ANALYSIS”

METHOD

1. State if your review was registered on PROSPERO or not.

2. Add a reason for why your start date for the search was January 1 2000 and not from the beginning of the databases collection, and in discussion, consider how this may have impacted your results/# of papers.

3. Line 112-113, pg 14: The sentence “Studies that included medical students within mixed populations were accepted if data specific to medical students could be extracted.” - was this for any proportion of medical students out of the whole sample of mixed populations? If so, this may have implications

4. Include metrics for inter-rater reliability between screeners. E.g. Kappa

5. Line 130, pg 14: remove capitalisation on “Data”

6. Line 144, pg 15: Add initials for quality reviewers.

7. Line 154-156, pg 15: “This model assumes that true prevalence varies across populations, which aligns with theoretical expectations about impostor phenomenon manifestation across different medical education contexts.” – add a reference for this theoretical expectations.

8. Line 160-162 – “While I² values above 75% conventionally indicate substantial heterogeneity, we interpreted this metric within the context of global prevalence studies where such values are normative rather than exceptional.” - add a reference for this approach , has this been used in other global prevalence reviews?

9. The terms “Sex” and “gender” are used interchangeably – please ensure consistency. If you are referring to biological sex, then use ‘sex’ and specify your variables.

10. Sensitivity analyses were mentioned in line 147 on pg 15 in the ‘ROB assessment’ section, but no description of these is given in the ‘Statistical analysis’ section. I am assuming that that was because all studies were low risk? If this is the case, state there was planned sensitivity analyses in the methods, but then clarify in results that they were not conducted and the reason.

11. Make it clearer in your methods, and then in your results (perhaps at line 222-3, pg 18) that you did one meta-analysis with CIPS studies, and another sub-group analysis with all YIS studies, and whether this was post-hoc or planned a priori? And that the findings you will be focusing on in your reporting will be of the CIPS pooled prevalence rates – you have made this clear in your abstract, perhaps reiterate that at a few points in your results where you state prevalence rates to remind the reader.

12. Line 165, pg 15 – define preclinical and clinical here with the years as you have done later in the results. Especially as it varies across countries, and can be confusing. You have addressed this difference in the discussion, which is great.

RESULTS

1. Line 180, pg 16: “due to non-corresponding populations of undergraduate medical students (n=88)” – please define this more clearly, I am unsure what this refers to.

2. In line 187, pg 16, its stated that the 6 studies using the YIS were excluded from the meta-analysis – firstly, include the 6 studies as citations. Secondly, clarify here that you did examine the YIS studies as a sub-group analysis, so that they were included just not in the ‘MAIN’ meta analysis. At first, I was a bit confused when you mentioned you excluded the YIS as its was a sclae mentioned in your eligibility criteria, then later when I saw the sub-group analysis of the 4 YIS studies, I realised what you had done, so perhaps this could be clarified a little better for the reader.

3. Include this sentence in the METHOD instead – line 236-239, pg 25: “The tool evaluated nine critical appraisal criteria: appropriateness of sampling frame, participant recruitment methods, sample size adequacy, detailed description of subjects and settings, data analysis coverage, validity of identification methods, standardized measurement, statistical analysis appropriateness, and response rate handling.”

4. Suggest adding an additional heading for “Qualitative results” at the start of the results, and then “Quantitative results” when you start reporting the results for only those studies included in the meta-analysis, as I got a little lost trying to determine which group of studies you were talking about at what point.

5. Line 240, pg 25: “All studies (100%) met criteria for using validated instruments (CIPS) ” – include N of studies, 100% is not clear. Also, you mentioned YIS as acceptable scale in your inclusion criteria, and its listed in your table 1 for a few included studies.

6. Line 264-5, pg 26: “Minor variations in cut-off points (≥61 or ≥63) were considered clinically equivalent” - consider adding a reference you can cite to validate this approach or another study using this approach as precdent?

7. In the subanalysis for YIS, state more clearly why only 4 studies were included and cite the 2 that were excluded and why.

DISCUSSION

1. Explanation of why you used the term phenomenon not syndrome should be moved to the introduction.

2. Line 386, pg 31: did you mean “clinically significant IP” or clinical levels of IP – also provide a ref for this claim that its clinically significant, especially as earlier it was stated it is not a diagnosable condition.

3. Good depth of consideration of the implications of the high heterogeneity on the results and our confidence in them.

4. Line 479-480, pg 35 – add that further research needs to be conducted in Peru and Africa to verify these findings and in Peru, to explore potentially protective factors.

5. Line 485-487, pg 35: “485 The persistence of high IP rates despite increased institutional attention to student wellbeing indicates that current approaches, primarily focused on individual resilience and stress management, may be insufficient.” - add citations for these current approaches and for the increased institutional awareness claim. Additionally, it strikes me that similar patterns are observed in mental health – despite increased awareness, rates are not going down. And we still largely provide individual-level interventions (see Petrie et al, 2025; . https://doi.org/10.1038/s44220-025-00450-2) despite major systemic issues.

6. Line 497, pg 36: Add a reference in the “professional identity formation” discussion.

7. Line 499 onwards, pg 36: Suggest in the discussion around co-morbidity with mental ill-health, another sentence or two highlighting the potential for reverse causation that cannot be addressed by cross-sectional designs – especially given that medical students experience higher rates of mental ill-health than other students.

8. Line 506, pg 36: add a reference for “contrasting with wider gender gaps in other populations” – and clarify which populations, and if only other student pops?

9. Line 519-20, pg 36: “The extremely high heterogeneity (I² = 97.08%) indicates substantial unexplained variation among studies.” - Add onto this sentence clearer qualification of your findings – this high heterogeneity means that we need to take these pooled rates with considerable caution. Include this in your concluding paragraph, and maybe abstract too.

10. Separate Future directions/recommendations from Conclusion into separate paragraphs and headings. Perhaps the first para of the conclusion/recommendations section and the last sentence included as the final last ‘Conclusions” para?

11. In the recommendations section, where you list the next steps for institutions,– are there any evidence-based strategies or interventions for IP in med students or other pops, that could also be cited as next steps, for universities or medical bodies?

**********

6. PLOS authors have the option to publish the peer review history of their article (what does this mean?). If published, this will include your full peer review and any attached files.

If you choose “no”, your identity will remain anonymous but your review may still be made public.

**Do you want your identity to be public for this peer review?**  For information about this choice, including consent withdrawal, please see our Privacy Policy.

Reviewer #1: No

Reviewer #2: No

Reviewer #3: No

Reviewer #4: **Yes:** Kelly Lee

Reviewer #5: No

**********

[NOTE: If reviewer comments were submitted as an attachment file, they will be attached to this email and accessible via the submission site. Please log into your account, locate the manuscript record, and check for the action link "View Attachments". If this link does not appear, there are no attachment files.]

To ensure your figures meet our technical requirements, please review our figure guidelines: https://journals.plos.org/plosone/s/figures

You may also use PLOS’s free figure tool, NAAS, to help you prepare publication quality figures: https://journals.plos.org/plosone/s/figures#loc-tools-for-figure-preparation.

NAAS will assess whether your figures meet our technical requirements by comparing each figure against our figure specifications.

Attachment Submitted filename: PLOS 1.docx

# Author comment: Author response to Decision Letter 1

**DOI:** 10.1371/journal.pone.0351713.r003

9 Jul 2026

Reviewer #1

Reviewer/editor comment: You switch between IP imposter phenomenon and IS imposter syndrome. An explanation is provided in the discussion. Would it be possible to use only IP? If not, consider moving this explanation up to the intro.

Response: Thank you for this observation. We agree that the terminology was inconsistent and that the conceptual explanation appeared too late in the manuscript.

Change made: We now use “IP” throughout the manuscript except when referring to terminology used by original studies, search strategies, or specific instruments. We moved the explanation to the Introduction and clarified that “impostor phenomenon” is preferred because it is not a formal psychiatric diagnosis and the term “syndrome” may be pathologizing.

Reviewer/editor comment: The section on study selection process is confusing. The 71 full text articles excluding 47 does not get to 35.

Response: Thank you for identifying this arithmetic inconsistency. We agree. The number of full-text exclusions was incorrect in the previous version.

Change made: We corrected the study selection paragraph and Figure 1. Seventy-one full-text articles were assessed; 36 were excluded; therefore, 35 studies were included in the qualitative synthesis. The full-text exclusion reasons are now reported as: failure to report IP prevalence data or sufficient data to calculate prevalence (n=9), use of nonvalidated instruments (n=10), and mixed populations without extractable medical-student-specific data (n=17).

Reviewer/editor comment: I am unclear why you included studies from 2016 forward but your metaregression only included 2018-2024?

Response: Thank you for noting this lack of clarity. We agree that the methods and results needed to specify the analytic subset used for meta-regression.

Change made: We clarified that the meta-regression was restricted to the primary CIPS-based quantitative synthesis. The earliest study eligible for that synthesis was published in 2018; therefore, the meta-regression covered 2018-2024, even though the qualitative synthesis included studies from 2016 onward.

Reviewer/editor comment: In the intro line 64 you state “reaching over 75% in certain educational contexts.” Adding a word or two to this sentence would help with clarity.

Response: Thank you for this suggestion. We agree that the phrase was vague.

Change made: The Introduction now specifies that these higher estimates occur in “competitive, high-stakes, and clinically supervised educational contexts.”

Reviewer/editor comment: I understand the importance of addressing well being also among physicians and leaders and note you highlighted the need for systems change.

Response: Thank you for this comment. We agree that the relevance of IP extends beyond individual student coping and should be interpreted in relation to educational systems.

Change made: We revised the Discussion and Future directions to frame institutional action more cautiously, emphasizing assessment practices, feedback culture, mentoring structures, and learning climate rather than making unsupported causal claims.

Reviewer/editor comment: I do not understand the wide heterogeneity documented in your study and will leave discussions about that to the statisticians.

Response: Thank you for raising this point. We agree that the high heterogeneity requires clearer interpretation for readers.

Change made: We revised the Methods and Discussion to explain that high I² is common in prevalence meta-analyses, that the pooled estimate should not be interpreted as a universal rate, and that subgroup analyses by sampling design, sex, academic stage, region, and country should guide interpretation.

Reviewer #2

Reviewer/editor comment: The impact of the study could be highlighted more effectively. I suggest revisiting the final sentence of the abstract and the final sentence of the introduction’s first paragraph.

Response: Thank you for this suggestion. We agree that the relevance of the study could be framed more clearly at key positions.

Change made: We revised the Abstract and Introduction to explain why medical students are vulnerable to IP and to emphasize that the study provides global CIPS-based prevalence estimates while requiring cautious interpretation because of heterogeneity.

Reviewer/editor comment: In the Introduction, it would be beneficial to briefly touch upon the conceptual nature of IS. Is it typically conceptualized as a stable, global personality trait or as a context-dependent state?

Response: Thank you for this important conceptual point. We agree that the manuscript needed this nuance.

Change made: We added text to the Introduction explaining that IP may show trait-like persistence in some individuals but may also be activated or intensified by high-stakes evaluative environments, professional identity transitions, and repeated comparison with high-performing peers.

Reviewer/editor comment: In the Methods section, you note that the work was not registered on a clinical trials registry. For systematic reviews, standard practice is registration in PROSPERO. Please state whether the review protocol was registered in PROSPERO. If not, acknowledge this as a limitation.

Response: Thank you for this correction. We agree that “clinical trial number: not applicable” did not adequately address systematic-review registration.

Change made: We replaced that statement with: “The review protocol was not prospectively registered in PROSPERO, and no registration number is available.” We also added the absence of prospective PROSPERO registration as a limitation.

Reviewer/editor comment: You acknowledge the exclusion of PsycINFO and provide a rationale. The argument would be strengthened if supported by evidence or data on database overlap or coverage.

Response: Thank you for this point. We agree that the previous wording was too confident. We did not want to imply that excluding PsycINFO was inconsequential.

Change made: We revised the Search strategy to state that PsycINFO was not searched because of limited institutional access, that this may have missed psychology-focused records, and that the limitation was mitigated by searching MEDLINE/PubMed, EMBASE, Scopus, Web of Science, and manually checking references. We added a methodological citation on database combinations and also acknowledged PsycINFO omission in the Limitations.

Reviewer #3

Reviewer/editor comment: This systematic review and meta-analysis is timely and addresses a pertinent issue. It was well conducted; however, the main challenge is the failure to register the protocol on PROSPERO, even though it is now too late to do so.

Response: Thank you for this comment. We agree. Because the review was not prospectively registered, we did not attempt retrospective registration or imply that the protocol had been prospectively registered.

Change made: We now explicitly state in Methods that the review protocol was not prospectively registered in PROSPERO and added this issue to the Limitations.

Reviewer #4

Reviewer/editor comment: General comment: consider reporting a prevalence rate for medical education programs based on graduate-level education versus direct entry from secondary education; consider reducing manuscript length; assess consistent use of IP and IS and run-on sentences.

Response: Thank you for these general comments. We partially agree. We agree that entry structure may be an important source of heterogeneity, but we did not conduct this subgroup analysis because program-entry structure was not consistently reported at the study level and country-level classification could misclassify mixed-pathway systems.

Change made: We added this issue to the Limitations. We also standardized terminology to IP, reorganized the Results using qualitative and quantitative synthesis headings, separated Future directions from Conclusions, and split or rewrote long sentences.

Reviewer/editor comment: Title: Based upon the conclusion, it seems that the authors are recommending both methodological changes as well as medical education reform. The title does not reflect these themes.

Response: Thank you for this observation. We partially agree. The title accurately reflects the design and primary objective of the manuscript, whereas the previous conclusion was broader than the evidence allowed.

Change made: We retained the title but narrowed the Conclusions and separated Future directions from Conclusions. The final conclusion now emphasizes prevalence, heterogeneity, standardized measurement, probabilistic sampling, longitudinal research, and rigorously evaluated interventions without overextending the title.

Reviewer/editor comment: Abstract, Introduction: consider adding one additional sentence to expand the background as to why medical students are an at-risk population.

Response: Thank you for this suggestion. We agree.

Change made: The Abstract and Introduction now specify that medical training combines repeated high-stakes assessment, competitive peer comparison, hierarchical clinical supervision, and rapid professional identity transition.

Reviewer/editor comment: Abstract, Objective: consider removing “undergraduate” as some included studies are conducted in the US where medical school is graduate education.

Response: Thank you for this important terminology correction. We agree.

Change made: We replaced “undergraduate medical students” with “pre-licensure medical students” in the Abstract, Methods, and relevant parts of the manuscript.

Reviewer/editor comment: Methodology: consider defining the CIPS scale to elaborate on why this IP measurement was chosen instead of alternative scales.

Response: Thank you for this suggestion. We agree that the analytic role of CIPS needed clarification.

Change made: We added that CIPS was treated as the primary instrument because it is the most frequently used IP measure in the included literature, uses a 20-item format, and provides conventional severity thresholds that allow more consistent prevalence synthesis. YIS and Leary scale studies are now summarized separately.

Reviewer/editor comment: Line 111: consider removing, repeated from line 100.

Response: Thank you for noting this repetition. We agree.

Change made: We revised the Selection criteria to avoid repeating the search date range and instead focused the section on eligibility criteria and synthesis grouping.

Reviewer/editor comment: Line 125: consider elaborating on examples of discrepancies.

Response: Thank you for this suggestion. We agree.

Change made: The Study selection process now specifies that discrepancies included disagreements about population eligibility, instrument validity, extractability of medical-student-specific prevalence, and compatibility of IP thresholds for quantitative synthesis.

Reviewer/editor comment: Results, Line 181: Should this be IP measurement?

Response: Thank you for catching this inconsistency. We agree.

Change made: We replaced “IS measurement” with “IP assessment” and standardized terminology throughout the manuscript.

Reviewer/editor comment: Results, Lines 184-187: Text does not match Figure 1. The text states that 35 studies met eligibility but Figure 1 states 24 studies in qualitative synthesis. Same for quantitative meta-analysis. Please correct.

Response: Thank you for identifying this inconsistency. We agree.

Change made: We corrected the study selection text and Figure 1. The revised PRISMA flow now reports 35 studies in qualitative synthesis, 26 studies in the primary CIPS meta-analysis, and 4 studies in the exploratory YIS synthesis.

Reviewer/editor comment: Results, Lines 205-207: consider separating into two sets of parentheses.

Response: Thank you for this formatting suggestion. We agree.

Change made: We revised the sentence describing study designs and sampling so that counts and percentages are clearer and not overloaded in a single parenthetical statement.

Reviewer/editor comment: Results, Line 216: reassess citation stating “unspecified in one study.”

Response: Thank you for this observation. We agree that the previous wording was unclear.

Change made: We revised the sentence to state that sex distribution was not reported in Clark (2024).

Reviewer/editor comment: Results, Line 245: consider elaborating or defining “inadequately handled response rates.”

Response: Thank you for this suggestion. We agree.

Change made: We now define this as response rates that were either not reported or not accompanied by an assessment of non-response bias, such as comparison between responders and non-responders or discussion of how low participation could affect prevalence estimates.

Reviewer/editor comment: Results, Line 351-352: consider changing to “studies in the Middle East...” “studies in Asia...” etc.

Response: Thank you for this wording suggestion. We agree.

Change made: We revised the meta-regression paragraph to use “studies conducted in North America,” “studies conducted in the Middle East,” and “studies conducted in Asia.”

Reviewer/editor comment: Discussion, Lines 376-380: lengthy sentence is unclear; may consider splitting into two sentences.

Response: Thank you for this comment. We agree.

Change made: We rewrote the relevant Discussion paragraph and split long sentences to improve readability.

Reviewer/editor comment: Discussion, Line 386: consider removing “clinically” given that IP is not a psychiatric condition.

Response: Thank you for this correction. We agree.

Change made: We replaced “clinically significant IP” and related phrasing with “threshold-defined IP” or “moderate-to-severe IP according to CIPS thresholds.”

Reviewer/editor comment: Discussion, Line 424: consider removing “policy makers, educators, and institutional leaders require” and changing to “quantitative benchmarks are necessary to justify resource allocation, design targeted interventions...”

Response: Thank you for this wording suggestion. We agree.

Change made: The sentence now states that quantitative benchmarks are necessary to justify resource allocation, design targeted interventions, and monitor wellness programs, while also emphasizing cautious interpretation due to heterogeneity.

Reviewer/editor comment: Discussion, Line 479: consider elaborating on unique features of Peruvian medical education.

Response: Thank you for this suggestion. We partially agree. We agree that the Peru finding requires discussion, but we avoided speculative claims about protective features without direct data.

Change made: We revised the paragraph to state that the lower prevalence observed in Peru may reflect sampling, institutional context, measurement implementation, cultural response patterns, or unmeasured educational factors. We also added that further studies in Peru are needed to determine reproducibility and explore potential protective factors.

Reviewer/editor comment: Conclusion: the conclusion does not match the title; the authors should consider narrowing the focus of the manuscript.

Response: Thank you for this comment. We agree that the previous conclusion was too broad.

Change made: We narrowed the final conclusion, separated Future directions from Conclusions, and framed educational reform as a future research and intervention priority rather than a direct causal inference from the meta-analysis.

Reviewer/editor comment: Table 1: Tables need footnotes to explain abbreviations used as well as scale for the risk of bias analysis.

Response: Thank you for this suggestion. We agree.

Change made: We added a Table 1 footnote defining CIPS, YIS, NR, IP, and the JBI/Munn risk-of-bias scoring range and categories.

Reviewer/editor comment: Table 1: In the Holliday (2019) study, was the data for medical students and dental medicine students separated for analysis?

Response: Thank you for raising this important point. We agree that this needed clarification.

Change made: We clarified in the Study selection section that some studies were retained for qualitative synthesis but were not included in the primary CIPS meta-analysis when pooling-compatible medical-student-specific prevalence data could not be extracted. Holliday was therefore not included in the primary CIPS quantitative synthesis.

Reviewer/editor comment: Figures: All figures are blurry and need high-resolution images.

R

Attachment Submitted filename: Response to Reviewers.docx

# Peer review: Decision Letter 1

**DOI:** 10.1371/journal.pone.0351713.r004

**Authors:** Diego A. Forero

21 Jul 2026

Global prevalence of impostor phenomenon in medical students: a systematic review and meta-analysis

PONE-D-25-65587R1

Dear Dr. Valladares-Garrido,

We’re pleased to inform you that your manuscript has been judged scientifically suitable for publication and will be formally accepted for publication once it meets all outstanding technical requirements.

Within one week, you’ll receive an e-mail detailing the required amendments. When these have been addressed, you’ll receive a formal acceptance letter and your manuscript will be scheduled for publication.

An invoice will be generated when your article is formally accepted. Please note, if your institution has a publishing partnership with PLOS and your article meets the relevant criteria, all or part of your publication costs will be covered. Please make sure your user information is up-to-date by logging into Editorial Manager at Editorial Manager® and clicking the ‘Update My Information' link at the top of the page. For questions related to billing, please contact billing support.

If your institution or institutions have a press office, please notify them about your upcoming paper to help maximize its impact. If they’ll be preparing press materials, please inform our press team as soon as possible -- no later than 48 hours after receiving the formal acceptance. Your manuscript will remain under strict press embargo until 2 pm Eastern Time on the date of publication. For more information, please contact onepress@plos.org.

Kind regards,

Diego A. Forero, MD; PhD

Academic Editor

PLOS One

Additional Editor Comments (optional):

Reviewers' comments:

Reviewer's Responses to Questions

**Comments to the Author**

1. If the authors have adequately addressed your comments raised in a previous round of review and you feel that this manuscript is now acceptable for publication, you may indicate that here to bypass the “Comments to the Author” section, enter your conflict of interest statement in the “Confidential to Editor” section, and submit your "Accept" recommendation.

Reviewer #4: All comments have been addressed

Reviewer #5: All comments have been addressed

**********

2. Is the manuscript technically sound, and do the data support the conclusions?

The manuscript must describe a technically sound piece of scientific research with data that supports the conclusions. Experiments must have been conducted rigorously, with appropriate controls, replication, and sample sizes. The conclusions must be drawn appropriately based on the data presented.

Reviewer #4: Yes

Reviewer #5: Yes

**********

3. Has the statistical analysis been performed appropriately and rigorously?

Reviewer #4: Yes

Reviewer #5: Yes

**********

4. Have the authors made all data underlying the findings in their manuscript fully available?

The PLOS Data policy requires authors to make all data underlying the findings described in their manuscript fully available without restriction, with rare exception (please refer to the Data Availability Statement in the manuscript PDF file). The data should be provided as part of the manuscript or its supporting information, or deposited to a public repository. For example, in addition to summary statistics, the data points behind means, medians and variance measures should be available. If there are restrictions on publicly sharing data—e.g. participant privacy or use of data from a third party—those must be specified.

Reviewer #4: Yes

Reviewer #5: Yes

**********

5. Is the manuscript presented in an intelligible fashion and written in standard English?

PLOS ONE does not copyedit accepted manuscripts, so the language in submitted articles must be clear, correct, and unambiguous. Any typographical or grammatical errors should be corrected at revision, so please note any specific errors here.

Reviewer #4: Yes

Reviewer #5: Yes

**********

6. Review Comments to the Author

Please use the space provided to explain your answers to the questions above. You may also include additional comments for the author, including concerns about dual publication, research ethics, or publication ethics. (Please upload your review as an attachment if it exceeds 20,000 characters)

Reviewer #4: (No Response)

Reviewer #5: (No Response)

**********

7. PLOS authors have the option to publish the peer review history of their article (what does this mean?). If published, this will include your full peer review and any attached files.

If you choose “no”, your identity will remain anonymous but your review may still be made public.

**Do you want your identity to be public for this peer review?**  For information about this choice, including consent withdrawal, please see our Privacy Policy.

Reviewer #4: No

Reviewer #5: No

**********

# Editor report: Acceptance letter

**DOI:** 10.1371/journal.pone.0351713.r005

**Authors:** Diego A. Forero

PONE-D-25-65587R1

PLOS One

Dear Dr. Valladares-Garrido,

I'm pleased to inform you that your manuscript has been deemed suitable for publication in PLOS One. Congratulations! Your manuscript is now being handed over to our production team.

At this stage, our production department will prepare your paper for publication. This includes ensuring the following:

* All references, tables, and figures are properly cited

* All relevant supporting information is included in the manuscript submission,

* There are no issues that prevent the paper from being properly typeset

You will receive further instructions from the production team, including instructions on how to review your proof when it is ready. Please keep in mind that we are working through a large volume of accepted articles, so please give us a few days to review your paper and let you know the next and final steps.

Lastly, if your institution or institutions have a press office, please let them know about your upcoming paper now to help maximize its impact. If they'll be preparing press materials, please inform our press team within the next 48 hours. Your manuscript will remain under strict press embargo until 2 pm Eastern Time on the date of publication. For more information, please contact onepress@plos.org.

You will receive an invoice from PLOS for your publication fee after your manuscript has reached the completed accept phase. If you receive an email requesting payment before acceptance or for any other service, this may be a phishing scheme. Learn how to identify phishing emails and protect your accounts at https://explore.plos.org/phishing.

If we can help with anything else, please email us at customercare@plos.org.

Thank you for submitting your work to PLOS ONE and supporting open access.

Kind regards,

PLOS ONE Editorial Office Staff

on behalf of

Dr. Diego A. Forero

Academic Editor

PLOS One
